# NRF2-mediated ferroptosis suppression defines a cancer-specific vulnerability in tumors

**DOI:** 10.1016/j.redox.2026.104352

**Published:** 2026-08-19

**Authors:** Dichun Huang, Mae Zhang, Ben N. Stansfield, Mona Ahmed, Tianbao Xu, Lewis Alexander, Annadurai Anandhan, Mengjiao Ma, Pengfei Liu, Yang Yang-Hartwich, Deyu Fang, Eli Chapman, Joe G.N. Garcia, Pak Kin Wong, Aikseng Ooi, Donna D. Zhang

**Affiliations:** a Department of Molecular Medicine, Center for Inflammation Science and Systems Medicine, UF Scripps Institute for Biomedical Innovation and Technology, Jupiter, FL, USA; b Department of Pharmacology and Toxicology, College of Pharmacy, University of Arizona, Tucson, AZ, USA; c Genetics Graduate Interdisciplinary Program, University of Arizona, Tucson, AZ, USA; d Department of Biomedical Engineering, The Pennsylvania State University, University Park, PA, USA; e Department of Pharmacology and Therapeutics, College of Medicine, University of Florida, Gainesville, FL, USA; f International Joint Research Center on Cell Stress and Disease Diagnosis and Therapy, The Second Affiliated Hospital of Xi'an Jiaotong University, Xi'an, China; g Department of Obstetrics, Gynecology, and Reproductive Sciences, Yale School of Medicine, New Haven, CT, USA; h Department of Pathology, Northwestern University Feinberg School of Medicine, Chicago, IL, USA; i University of Florida Health Cancer Center, University of Florida, Gainesville, FL, USA; j Division of Hematology and Oncology, Department of Medicine, College of Medicine, University of Florida, Gainesville, FL, USA; k Department of Medicinal Chemistry, University of Florida, Gainesville, FL, 32610, USA

## Abstract

NRF2 is a master regulator of redox and metabolic homeostasis that protects normal tissues from stress but is frequently hijacked by cancers to sustain survival and therapy resistance. Although NRF2 is dispensable for normal tissue function, its role in maintaining cancer cells within the native tumor microenvironment has remained undefined. Here, we uncover an essential and previously unrecognized tumor-specific dependency on NRF2. Using an inducible *Kras*^*FSF.G12D/+*^;*Nrf2*^*Fl/Fl*^;*Rosa26*^*CreERT2/CreERT2*^ (KNR) mouse lung cancer model, we demonstrate that *NRF2* deletion alone, without pharmacologic intervention, eradicates cancer cells, reduces tumor burden, and prolongs survival. Single-cell RNA sequencing coupled with artificial intelligence–based genotype classification revealed that *NRF2*-deleted cancer cells are selectively eliminated, whereas non-cancerous cells tolerate *NRF2* loss. Mechanistically, *NRF2* deletion induces ferroptosis, a regulated iron-dependent cell death pathway, evidenced by induction of canonical ferroptotic genes (*Ptgs2*, *Acsl4*, *Tfrc*) and protein markers (SO_2_/_3_-PRDX3, COX2, TfR1). Importantly, these data support that *NRF2* loss induces ferroptotic cell death *in vivo* within established tumors, in the absence of exogenous ferroptosis inducers or external stress. These findings establish that cancer cells depend on NRF2 to suppress intrinsic ferroptotic stress for survival, a dependency not shared by normal tissues. This discovery fundamentally redefines the pathological role of NRF2 and positions NRF2 inhibition as a standalone, tumor-selective therapeutic strategy to eliminate Kras-driven malignancies by unleashing ferroptosis.

## Teaser

NRF2 protects normal tissues, yet is frequently activated in cancer, where it promotes survival and therapy resistance. Whether NRF2 is required for maintenance of established tumors *in vivo* has remained unresolved. Using an inducible genetic model allowing deletion of *NRF2* after tumor formation, we show that NRF2 is dispensable in normal cells but essential for the survival of KRAS-driven cancer cells, revealing a tumor-specific dependency that creates a therapeutic window for NRF2 inhibition. Mechanistically, we demonstrate that cancer cells rely on NRF2 to continuously suppress ferroptotic stress, even in the absence of exogenous ferroptosis inducers. These findings establish a physiological role for ferroptosis as an intrinsic barrier to tumor maintenance and identify NRF2 inhibition as a potential standalone, tumor-selective therapeutic strategy.

## 1 Introduction

NRF2 is a transcription factor that orchestrates cellular defense mechanisms against oxidative stress, proteotoxicity, metabolic dysregulation, and iron-overload by activating a set of antioxidant response element (ARE)-bearing genes [[1], [2], [3]]. In normal tissues, NRF2 is ubiquitously expressed but maintained at low basal levels through its interaction with KEAP1, which directs NRF2 for ubiquitination and subsequent degradation via the KEAP1-CUL3-RBX1 E3 ubiquitin ligase complex [[4], [5], [6], [7], [8]]. The ability of NRF2 to be readily activated by chemopreventive compounds makes it an attractive target for cancer prevention [9,10]. However, it has become clear that cancer cells exploit this protective mechanism to support their survival. *KEAP1/NRF2* mutations are observed in approximately 30% of non-small cell lung cancer (NSCLC) patients, leading to NRF2 stabilization and constitutive activation [[11], [12], [13]]. High levels of NRF2 upregulate the expression of genes that promote cell survival and proliferation, ultimately contributing to tumor progression and metastasis, cancer-therapy resistance, and poor prognosis observed in lung cancer patients [[14], [15], [16], [17], [18], [19]].

Ferroptosis, a regulated form of cell death distinct from apoptosis and necrosis, is driven by iron-dependent lipid peroxidation [20,21]. Ferroptosis has also been proposed to function as an endogenous tumor-suppressive mechanism, but clinically detectable tumors may evade this barrier through adaptive survival programs, including activation of NRF2-dependent antioxidant and iron-homeostatic pathways [22,23]. Although the importance of ferroptosis in pathological and physiological contexts remains unclear, inducing ferroptosis holds promise as a therapeutic strategy to eliminate apoptosis-resistant cancer cells [24]. However, NRF2 has emerged as a critical suppressor of ferroptosis by upregulating multiple target genes that encode proteins involved in inhibiting lipid peroxidation and maintaining iron homeostasis [25,26]. These include SLC7A11, which facilitates cystine import for glutathione synthesis, and GCLC and GCLM, which catalyze the rate-limiting step of glutathione production. Glutathione serves as a cofactor for GPX4, the key anti-ferroptosis enzyme that reduces lipid hydroperoxides. Additionally, NRF2 upregulates ferroptosis suppressor protein 1 (FSP1), which regenerates coenzyme Q10 (CoQ10) into its antioxidant form, ubiquinol, and AKR1C1-3, which degrades lipid peroxides generated by lipoxygenases. In addition to regulating lipid peroxidation, NRF2 maintains iron homeostasis by inducing the expression of ferritin heavy and light chains (FTH1 and FTL), which sequester excess labile iron. NRF2 also regulates iron homeostasis by controlling the ferritinophagy process through distinct iron-dependent pathways, including HERC2-FBXL5-IRP1/2–mediated ferritin synthesis, HERC2-NCOA4–driven autophagosome recruitment of ferritin, and TFEB-VAMP8–regulated autophagosome-lysosome fusion for ferritin degradation. Additionally, NRF2 upregulates ferroportin (FPN1), the only known cellular iron exporter, thereby reducing the labile iron pool (LIP) [1].

Despite extensive investigation of NRF2 activation and KEAP1 loss in cancer, a fundamental question has remained unresolved: whether NRF2 is required for the survival of established tumors, as opposed to its well-characterized roles during tumor initiation and normal tissue protection. This question has been particularly difficult to address because NRF2 exerts distinct and opposing functions at different stages of tumorigenesis. In whole-body *Nrf2* knockout mouse models, loss of *NRF2* compromises cellular detoxification, redox balance, and genomic integrity in normal tissues, thereby enhancing tumor initiation. In contrast, in established tumors, NRF2 activation, most commonly driven by *KEAP1* or *NRF2* mutations, promotes tumor growth, survival, metabolic adaptation, and therapy resistance. Consequently, existing whole-body *Nrf2* and *Keap1* genetically engineered mouse models, in which NRF2 activity is altered globally or during development, inevitably conflate NRF2's tumor-suppressive role in normal tissues during tumor initiation with its tumor-promoting role in established cancers, precluding definitive assessment of NRF2 dependency in pre-existing tumors.

Our previous studies have demonstrated that inhibiting NRF2 enhances the efficacy of ferroptosis inducers both *in vitro* and *in vivo* [26]. Additionally, we observed that human cancer cells with *NRF2* deletion exhibit growth defects in 3D cultures or in immunodeficient mice (Supplementary Fig. S1) even in the absence of ferroptosis inducers [26], despite the tolerance of *Nrf2* deletion in whole-body *Nrf2* knockout mice under normal physiological conditions. This dichotomy supports our hypothesis that *Nrf2* deletion selectively impacts cancer cells while sparing non-cancerous cells, with cancer cells succumbing to ferroptosis due to their heightened metabolic and oxidative stress. To directly test whether NRF2 is required for the survival of established tumors, we established an inducible *Nrf2* deletion system in a *Kras*^*G12D*^-driven lung cancer model that enables *NRF2* ablation after tumor initiation and demonstrate, at single-cell resolution, that persistent NRF2-mediated suppression of ferroptosis is essential for cancer cell survival and tumor maintenance. These findings underscore the indispensable role of NRF2 in tumor maintenance and demonstrate that its inhibition is sufficient to eradicate cancer cells via ferroptosis, without the need for additional therapeutic agents. This discovery positions NRF2 inhibition as a standalone, paradigm-shifting approach for cancer treatment.

## 2 Results

### 2.1 TAM-induced *Nrf2* deletion reduces tumor burden and improves survival in KNR mice bearing *Kras*^*G12D*^-induced lung tumors

To investigate the role of NRF2 in tumor growth, we employed CRISPR-based techniques to generate A549 and SKOV3 cell lines with heterozygous (Het) or homozygous (KO) deletions of *NRF2*. While both *NRF2* Het and KO cell lines displayed comparable proliferation rates in 2D culture conditions, their capacity to form sizable organoids was impaired. In immunodeficient NSG (NOD scid gamma) mice, tumors derived from *NRF2* Het cells exhibited slower growth compared to those from WT cells in both A549-and SKOV3-derived lines (Supplementary Fig. S1A for A549, Fig. S1B for SKOV3). Notably, all *NRF2* KO cell lines were unable to develop into sizable tumors *in vivo* (Supplementary Fig. S1A for A549, Fig. S1B for SKOV3).

**Fig. 1:**
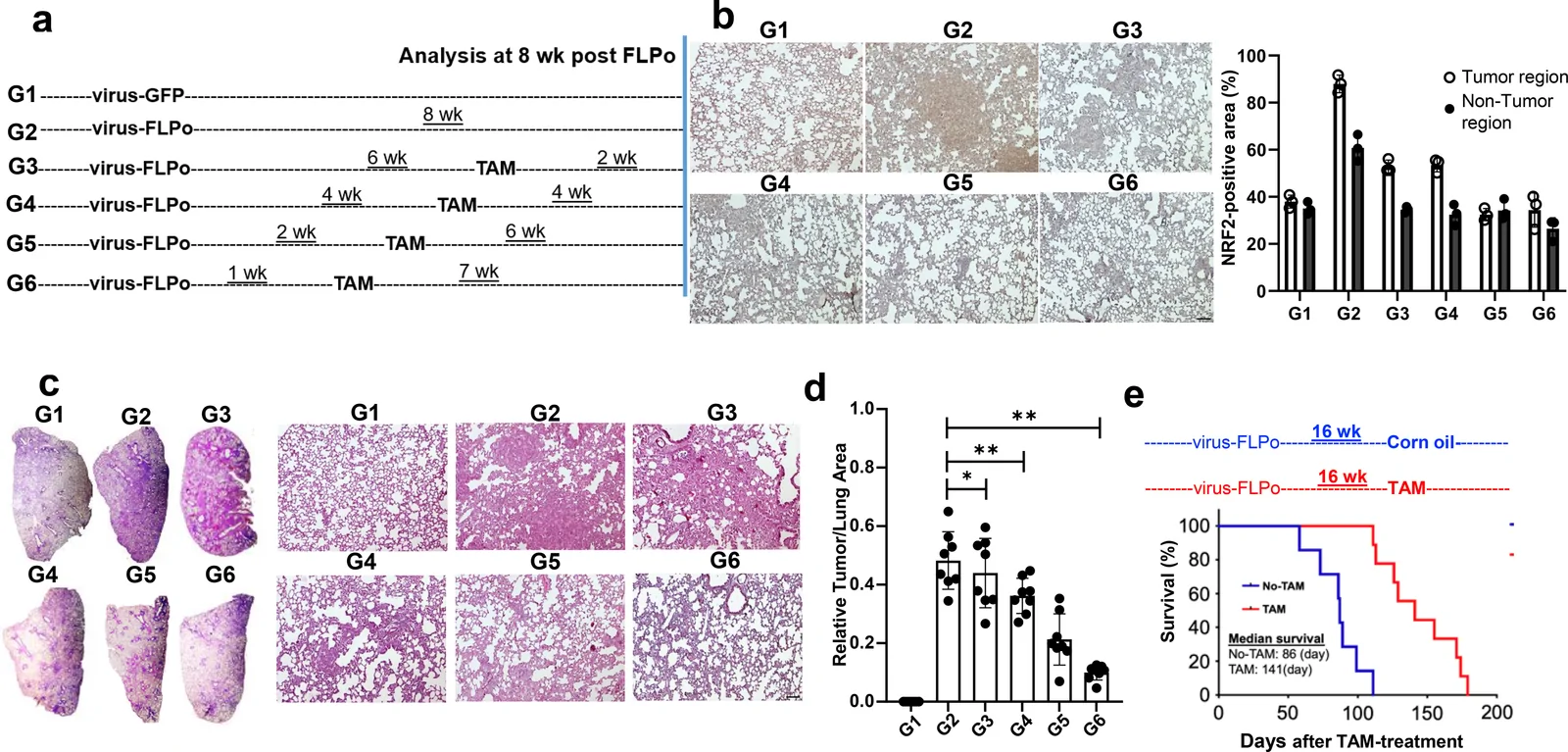
**TAM-induced *Nrf2* deletion reduces tumor burden and improves survival in KNR mice bearing *Kras*^*G12D*^-induced lung tumors. a**, Eight-week-old KNR mice were instilled with FlpO virus (2.75 × 10^8^ pfu) to activate *Kras* for tumor initiation (Groups 2–6) or with GFP virus as a control (Group 1). All Groups 2–6 received FlpO virus at the same time point and were analyzed 8 weeks after FlpO instillation; the only variable was the timing of TAM administration. Tamoxifen (TAM, 100 mg/kg body weight, daily for 5 days) was administered intraperitoneally (i.p.) at 6, 4, 2, or 1 weeks post-FlpO instillation (Groups 3–6, respectively), or not administered in Group 2. Lungs were harvested 8 weeks post-FlpO instillation for analysis. **b,** Immunohistochemistry (IHC) for NRF2 in lung tissues showing reduced NRF2 expression following TAM treatment. NRF2-positive areas were quantified separately in tumor and non-tumor regions. Scale bar, 100 μm. **c,** Hematoxylin and eosin (H&E)–stained lung sections showing adenomatous tumor morphology. Representative full-section images illustrate the overall distribution of tumor lesions within the lung, with higher-magnification views highlighting tumor architecture. Scale bar, 100 μm. **d**, Quantification of tumor burden as Relative Tumor/Lung Area (ratio of tumor area to total lung area) from H&E-stained slides. n = 8 per condition, two-tailed *t*-test, * indicate < 0.05, ** < 0.01. Statistical significance was determined by two-tailed *t*-test. *P < 0.05, **P < 0.01. **e,** Kaplan-Meier survival analysis of KNR mice instilled with FlpO virus at 8 weeks of age. Mice received either corn oil as a vehicle control (No-TAM) or TAM treatment starting 16 weeks post-FlpO instillation. Median survival times: No-TAM group = 86 days, TAM group = 141 days, n = 10 per group.

To further decipher the precise role and underlying mechanisms of NRF2 in tumor progression within the tumor microenvironment, we generated a conditional *Nrf2* knockout mouse model in a *Kras*^*G12D*^-driven tumor system: *Kras*^*FSF.G12D/+*^;*Nrf2*^*Fl/Fl*^;*Rosa26*^*CreERT2/CreERT2*^ (KNR) mice. This model system provides temporal control over both tumor initiation and *Nrf2* deletion, enabling precise assessment of NRF2's role in cancer progression after tumor initiation. As illustrated in Fig. 1A, 8-week-old mice were instilled with FlpO virus to activate *Kras*^*G12D*^ for tumor initiation (Groups 2-6), followed by tamoxifen (TAM) injection (Groups 3–6). TAM treatment effectively reduced NRF2 levels, as confirmed by immunohistochemistry (Fig. 1B). TAM-mediated *Nrf2* deletion led to a time-dependent decrease in tumor burden, with the earliest *Nrf2* deletion (Group 6, TAM at 1 week post-FlpO instillation) showing the most significant reduction in tumor burden (Fig. 1C and D). Representative full-section IHC images illustrate the overall distribution of adenomatous lesions within the lung and distinguish tumor regions from surrounding normal parenchyma (Fig. 1C). Consistent with prior reports, tumors in KrasG12D-only lung cancer models uniformly exhibit adenomatous histology and do not progress to invasive adenocarcinoma in the absence of additional cooperating mutations, such as *Trp53* loss [27]. Furthermore, TAM injection at 16 weeks post-FlpO instillation significantly increased the median survival from 86 days in the no-TAM group to 141 days in the TAM-treated group (Fig. 1E). Because *Nrf2* deletion is induced after tumor establishment in this model, these results isolate the role of NRF2 in tumor maintenance, independent of its previously described functions in tumor initiation or normal tissue protection.

### 2.2 TAM-induced *Nrf2* deletion alters lung cell populations in a time-dependent manner

**Fig. 2:**
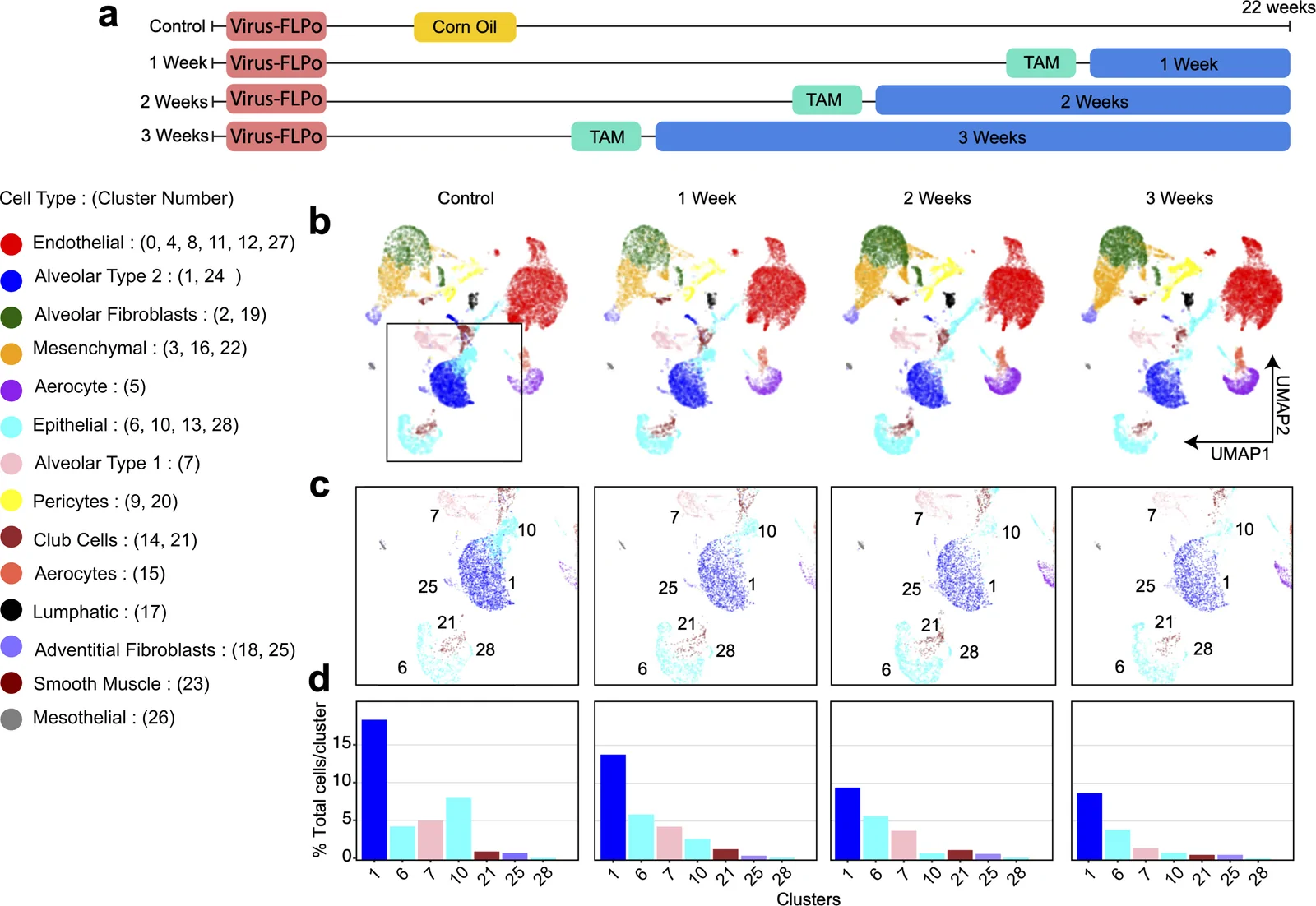
**TAM-induced *Nrf2* deletion alters lung cell populations in a time-dependent manner. a,** Experimental timeline illustrating the treatment groups. Control mice (Group 1) were instilled with FlpO virus but received corn oil instead of TAM. Groups 2–4 received FlpO virus for tumor initiation and were administered tamoxifen (TAM) (100 mg/kg body weight, i. p., daily for five days) at 1, 2, or 3 weeks before lung tissue harvesting at 22 weeks **b,** UMAP visualization of single-cell RNA sequencing (scRNA-seq) data showing lung cell populations across the four TAM treatment time points (Control, 1 Week, 2 Weeks, and 3 Weeks post-TAM treatment). Each point represents one cell. Cell types are annotated by color according to the legend, including endothelial, alveolar type 1 and type 2, fibroblast, mesenchymal, ependymal, epithelial, pericyte, club, aerocyte, lymphatic, adventitial fibroblast, smooth muscle, and mesothelial populations. **c,** Enlarged UMAP plots highlight specific clusters of interest (clusters 1, 6, 7, 10, 21, 25, and 28) across conditions. Numerical labels indicate Seurat cluster IDs, including clusters 1, 6, 7, 10, 21, 25, and 28. Cluster identities correspond to the color-coded cell-type annotations shown in b. **d,** Quantification of cell type distributions within the clusters across four groups. Percentages of total cells per cluster are plotted, showing a time-dependent decrease of the clusters in cell populations following TAM-induced *Nrf2* deletion. ScRNA-seq was performed on pooled lung tissues from five mice per treatment group; each time point represents one pooled dataset.

**Fig. 3:**
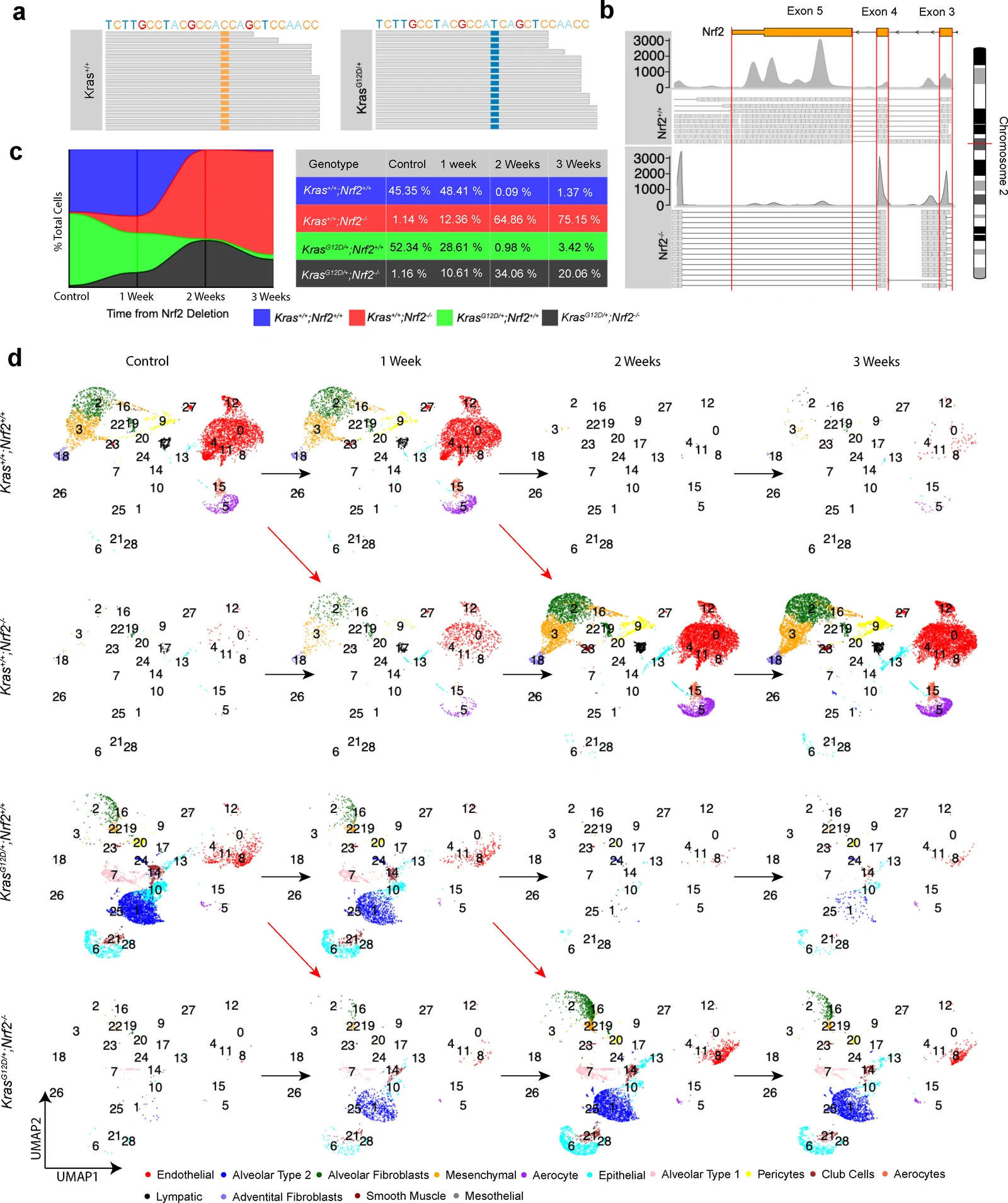
***Kras*-genotype-specific cell death following TAM-induced *Nrf2* deletion in KNR mice.** Panels A-B show the strategy used to identify Kras and Nrf2 genotypes from scRNA-seq data. Panel C summarizes temporal changes in the four resulting genotypes following TAM treatment, and Panel D visualizes their distribution within lung cell populations using UMAP analysis. **a,** Alignment of *Kras* wild-type (*Kras*^*+/+*^*)* and mutant (*Kras*^*G12D/+*^*)* sequences from scRNA-seq data. **b,** Coverage and read alignment of exons 3–5 of the *Nrf2* gene used for identification of *Nrf2*^*+/+*^ and *Nrf2*^*−/−*^ cells. Orange vertical lines denote exon boundaries. **c,** Alluvial plot visualizing the distribution of four genotypes (*Kras*^*+/+*^;*Nrf2*^*+/+*^, *Kras*^*+/+*^;*Nrf2*^*−/−*^, *Kras*^*G12D/+*^;*Nrf2*^*+/+*^, and *Kras*^*G12D/+*^;*Nrf2*^*−/−*^) across four time points. Numerical values indicate percentages of total cells for each genotype. **d,** UMAP visualization of scRNA-seq data showing lung cell populations/clusters stratified by Kras/Nrf2 genotype and treatment time point. Each point represents one cell. Numerical labels indicate Seurat cluster IDs, and colors denote annotated cell types according to the legend, consistent with the cell-type annotations in Fig. 2. Red arrows highlight temporal shifts in selected genotype-defined cell populations/clusters following TAM-induced *Nrf2* deletion. Same scRNA-seq dataset as described in Fig. 2.

**Fig. 4:**
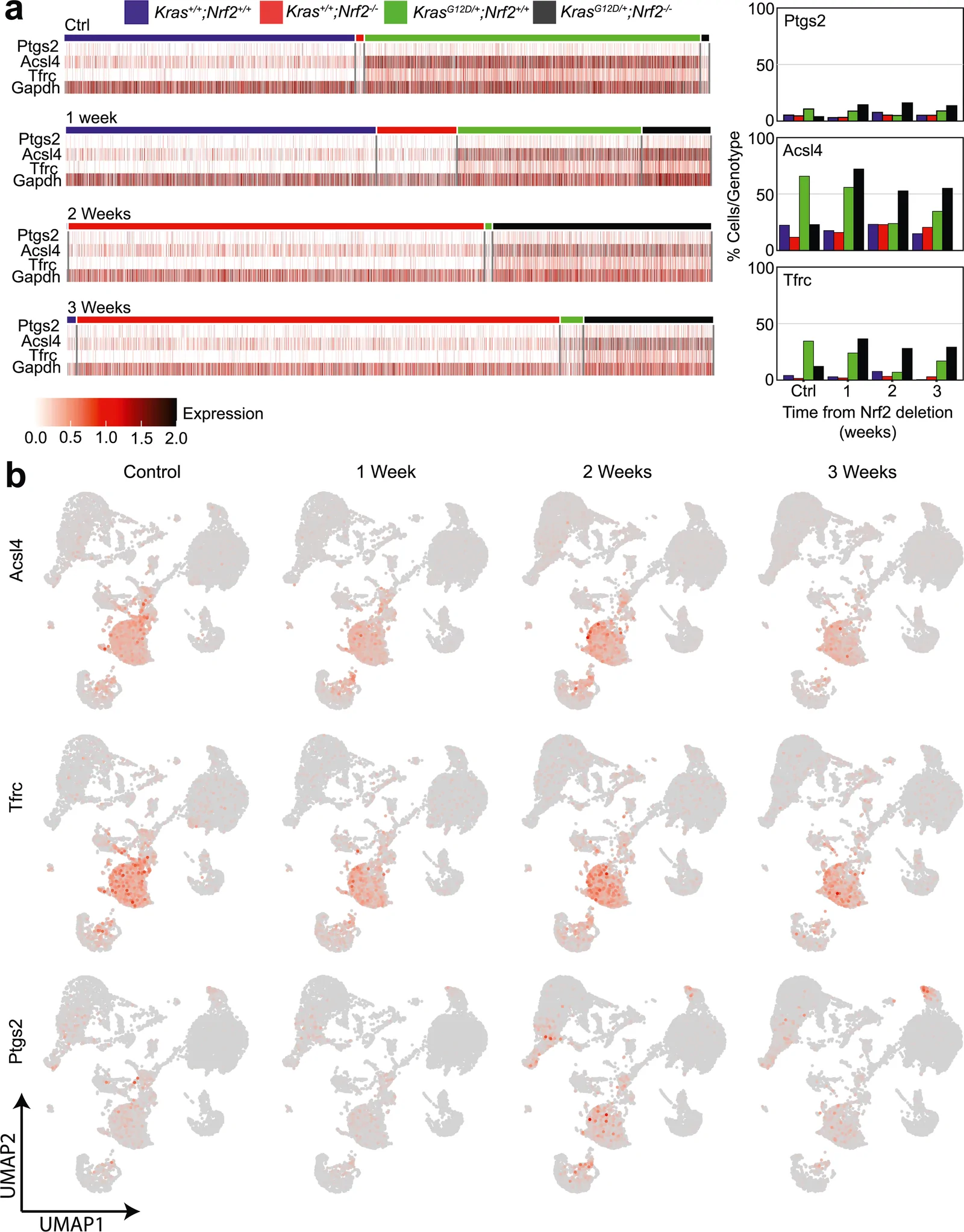
***Nrf2* deletion induces ferroptosis-related gene expression in *Kras*^*G12D*^ cancer cells. a,** Heatmap depicting the expression levels of *Ptgs2*, *Acsl4*, and *Tfrc* across genotypes (*Kras*^*+/+*^;*Nrf2*^*+/+*^, *Kras*^*+/+*^;*Nrf2*^*−/−*^, *Kras*^*G12D/+*^;*Nrf2*^*+/+*^, and *Kras*^*G12D/+*^;*Nrf2*^*−/−*^) over time following TAM-induced *Nrf2* deletion. Expression levels are normalized, and color intensity indicates relative expression. Bar graphs quantify the percentage of cells per genotype expressing *Ptgs2*, *Acsl4*, and *Tfrc* at each time point (Control, 1, 2, and 3 weeks post-*Nrf2* deletion). **b,** UMAP projections showing the spatial distribution of *Ptgs2*, *Acsl4*, and *Tfrc* expression across single-cell clusters at each time point. Red cells indicate high expression levels, highlighting ferroptosis-related activity. Same scRNA-seq dataset as described in Fig. 2.

**Fig. 5:**
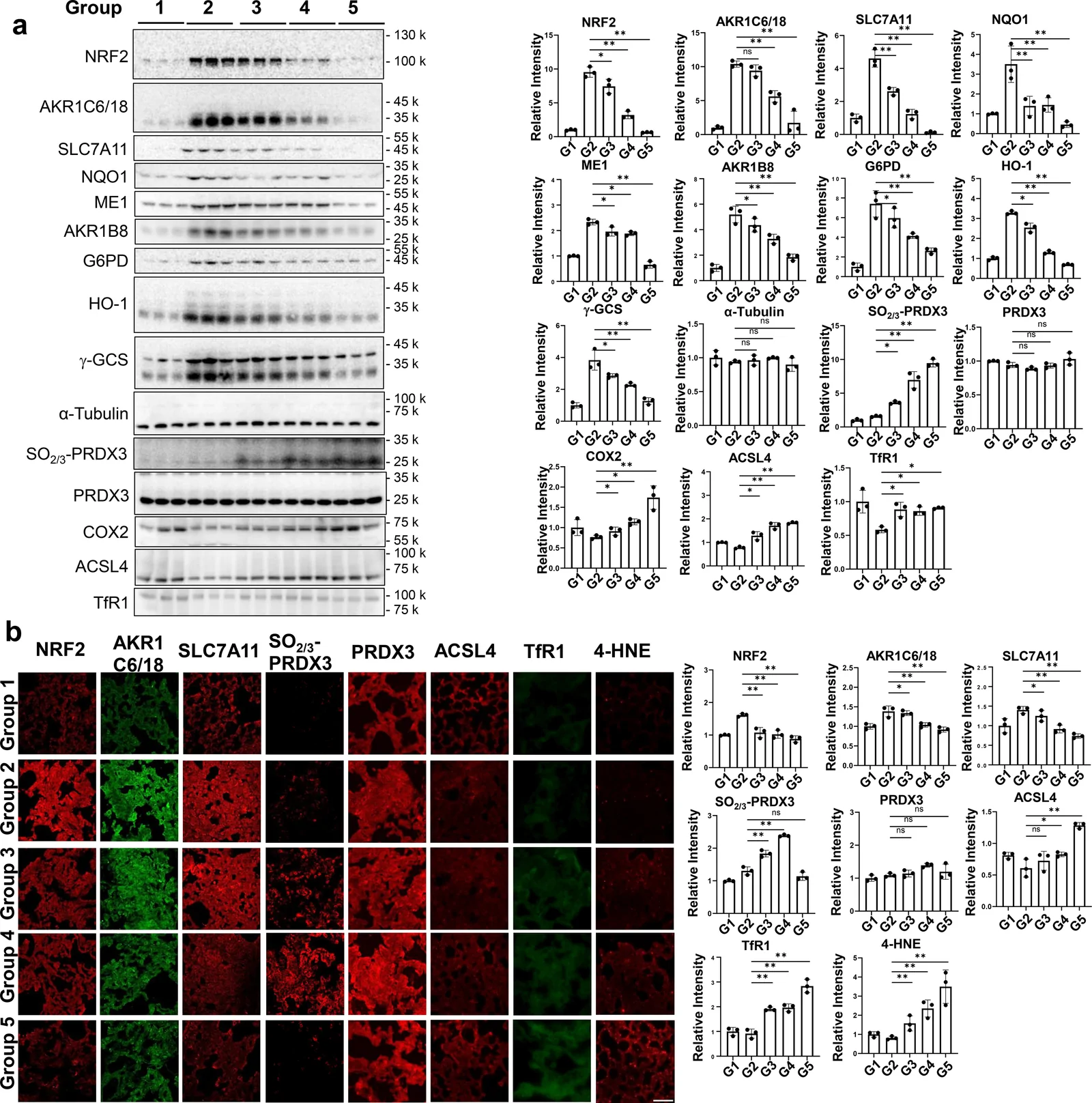
***Nrf2* deletion in *Kras*^*G12D*^ tumors promotes ferroptosis**. Group 1-5 described in Fig. 1: no tumor (group 1), no-TAM (group2), TAM injection for 2 weeks (group 3), 4 weeks (group 4), and 6 weeks (group 5). **a,** Immunoblot analysis of lung tissue lysates from KNR mice bearing *Kras*^*G12D*^-induced lung tumors across time points following TAM treatment for NRF2, its target genes encoding AKR1C6/18, SLC7A11, NQO1, ME1, AKR1B8, G6PD, HO-1, and γ-GCS, and ferroptosis-associated markers (SO_2/3_-PRDX3, PRDX3, COX2, ACSL4, and TfR1). α-Tubulin was used as the loading control, and total PRDX3 was included as a control for SO_2/3_-PRDX3. Note: Group 6 from Fig. 1 was not included in this analysis. Densitometric quantification and statistical analysis of the immunoblot data shown in Fig. 5A are presented to the right. Protein expression was normalized to α-tubulin and quantified by densitometry. Data are presented as mean ± SD (n = 3 biological replicates per group). TAM-induced *Nrf2* deletion resulted in progressive reductions in NRF2 and its downstream target proteins, including AKR1C6/18, SLC7A11, NQO1, ME1, AKR1B8, G6PD, HO-1, and γ-GCS. In contrast, ferroptosis-associated markers, including SO2/3-PRDX3, COX2, ACSL4, and TfR1, increased after Nrf2 deletion, whereas total PRDX3 remained unchanged. Statistical significance was determined by one-way ANOVA followed by Tukey's multiple-comparison test. *P < 0.05, **P < 0.01; ns, not significant. **b,** Immunofluorescence analysis of these lung tissue sections was performed for NRF2, its target genes encoding AKR1C6/18 and SLC7A11, and the ferroptosis marker SO_2/3_-PRDX3, with PRDX3 as a control. SO_2/3_-PRDX3 and total PRDX3 images are shown from serial (consecutive) sections, ACSL4, TfR1, and 4-HNE. Scale bar, 50 μm. Quantification of immunofluorescence signal intensity from panel b is shown to the right. Fluorescence intensities of the indicated NRF2 target proteins and ferroptosis-associated markers were quantified from representative images and normalized to the corresponding control group (Group 2). Data are presented as mean ± SD (n = 3 biological replicates). Statistical significance was determined by one-way ANOVA followed by Tukey's multiple-comparison test. *P < 0.05, **P < 0.01; ns, not significant.

To evaluate the effects of TAM-induced *Nrf2* deletion on lung cellular composition, single-cell RNA sequencing was performed on lungs harvested at multiple time points following tamoxifen administration. (Fig. 2A). This scRNA-seq experiment was performed in a separate cohort from the 8-week endpoint experiments shown in Fig. 1, Fig. 5. For Fig. 2, Fig. 3, Fig. 4, lungs were harvested 22 weeks after FlpO-mediated tumor initiation, and tamoxifen was administered 1, 2, or 3 weeks before tissue collection to enable genotype-resolved analysis of tumor and non-tumor cell populations in lungs with sufficient tumor burden. Lungs from five mice per treatment group were pooled before cell isolation and library preparation; therefore, each time point represents a single pooled dataset, and the resulting UMAP, genotype-distribution, and gene-expression analyses are descriptive rather than statistically powered comparisons across independent biological replicates. Uniform Manifold Approximation and Projection (UMAP) revealed distinct lung cell populations, with clear shifts in cell-type distributions following *Nrf2* deletion (Fig. 2B). As the duration of TAM treatment increased, alveolar type 2 cells (blue, cluster 1) and epithelial cells (cyan, cluster 10) decreased, whereas mesenchymal cells (orange) and endothelial cells (red) increased. Consistent with these global shifts, the enlarged UMAP views showed reduced representation of clusters 1, 7, and 10. Alveolar type 2 cells (cluster 1), alveolar type 1 cells (cluster 7), and epithelial cells (cluster 10) progressively decreased following TAM treatment (Fig. 2C). By 3 weeks post-TAM treatment, a near-complete depletion of specific clusters (7 and 10) was observed (Fig. 2C), consistent with pronounced changes in epithelial cell representation following *Nrf2* deletion. Quantification of cell distributions (Fig. 2D) further supports these observations, with a time-dependent decrease in alveolar type 1 and type 2 cells (cluster 1 and 7), as well as epithelial cells (clusters 10). These analyses reveal dynamic changes in cellular composition following *Nrf2* deletion.

### 2.3 *Nrf2* deletion leads to *Kras*-genotype-specific cell death

To evaluate the effects of TAM-induced *Nrf2* deletion in *Kras*^*+/+*^ (non-cancerous) and *Kras*^*G12D/+*^ (cancer) cells, we analyzed scRNA-seq data of KNR mouse lung tissues across four time points. Because accurate identification of the four genotypes (*Kras*^*+/+*^;*Nrf2*^*+/+*^, *Kras*^*+/+*^;*Nrf2*^*−/−*^, *Kras*^*G12D/+*^;*Nrf2*^*+/+*^, *Kras*^*G12D/+*^;*Nrf2*^*−/−*^) was complicated by the sparse and variable coverage inherent to single-cell RNA sequencing, we trained several binary classification neural network models and **combined their outputs through** an ensemble learning approach. Fig. 3 presents this analysis sequentially: panels A and B establish the basis for genotype classification, panel C summarizes temporal changes in predicted genotype distributions, and panel D shows how these predicted genotypes are distributed across lung cell populations. Because these assignments were inferred from sparse single-cell transcriptomic data, they should be interpreted as model-based genotype predictions rather than definitive per-cell genotype calls.

The training sets for the models utilized cells with complete alignment of sequencing reads to the *Kras* locus, allowing clear distinction between *Kras*^*+/+*^ and *Kras*^*G12D/+*^(Fig. 3A), and *Nrf2* exon 5 coverage that reliably differentiated *Nrf2*^*+/+*^ from *Nrf2*^*−/−*^ cells (Fig. 3B). The inferred genotype assignments were subsequently evaluated using exon-specific read coverage, temporal recombination patterns, and independent biological validation throughout the study.

The genotypes predicted by the binary classification neural network models revealed progressive and distinct shifts in cellular genotypes over time following TAM-induced *Nrf2* deletion (Fig. 3C). In the *Kras*^*+/+*^ background, representing non-cancerous cells, the proportion of *Nrf2*^*−/−*^ cells progressively increased, reaching 75.15% by three weeks, compared to 12.36% at one week (in red), whereas the proportion of *Nrf2*^*+/+*^ cells progressively decreased, reaching background levels by two weeks (in blue), demonstrating the efficiency of TAM-mediated *Nrf2* deletion. This result suggests that non-cancerous cells tolerate TAM-mediated *Nrf2* deletion. Conversely, in the *Kras*^*G12D/+*^ background (cancer cells), the *Nrf2*^*−/−*^ population initially increased, rising from 10.61% at one week to 34.06% at two weeks, but further to 20.06% at three weeks (Fig. 3C, in black). This pattern suggests an initial increase followed by partial elimination of *Kras*^*G12D/+*^;*Nrf2*^*−/−*^ cells between weeks two and three. In contrast, *Kras*^*G12D/+*^;*Nrf2*^*+/+*^ cells exhibited a consistent decrease starting at 52.34% at control and progressively decreased to zero by two weeks (Fig. 3C, in green), attributed to the efficiency of TAM-mediated *Nrf2* deletion. When the changes of *Kras*^*+/+*^;*Nrf2*^*−/−*^ vs. *Kras*^*G12D/+*^;*Nrf2*^*−/−*^ cell populations were compared from the time of peak *Nrf2* deletion (two weeks) to three weeks, the percentage of *Kras*^*+/+*^;*Nrf2*^*−/−*^ cells (non-cancerous cells) continued to increase (from 64.86% to 75.15%, Fig. 3C, in red), while that of *Kras*^*G12D/+*^;*Nrf2*^*−/−*^ cells (cancer cells) decreased (from 34.06% to 20.06%, Fig. 3C, in black). These results indicate that NRF2 is essential for *Kras*^*G12D*^-driven cancer cell survival, whereas it is dispensable for non-cancerous cell survival. These *Kras*-genotype-specific population dynamics underscore NRF2's divergent roles in sustaining cell survival in non-cancerous versus cancer cells. The UMAP dimensionality reduction of scRNA-seq data (Fig. 3D) demonstrated distinct cell clusters and their temporal shifts in cell populations/clusters across the four genotypes following TAM-induced *Nrf2* deletion. The red arrows highlight changes in specific clusters, in relation to genotype changes following TAM-induced *Nrf2* deletion. After one week following TAM treatment, nearly 50% of cell populations in clusters distributed to both *Nrf2*^*+/+*^ and *Nrf2*^*−/−*^ genotypes in either *Kras*^*+/+*^ or *Kras*^*G12D/+*^ background. However, in non-cancerous *Kras*^*+/+*^ cells, the cell populations/clusters drastically shifted to *Nrf2*^*−/−*^ cells by two and three weeks (Fig. 3D), indicating the dominance of *Kras*^*+/+*^;*Nrf2*^*−/−*^ cells (Fig. 3C and 64.86% at 2 weeks and 75.15% at 3 weeks, in red). In contrast, in *Kras*^*G12D/+*^ cells, the population/clusters shifting to *Nrf2*^*−/−*^ peaked at two weeks (Fig. 3C and 34.06%, in black, Fig. 3D), but declined by three weeks (Fig. 3C and 20.06%, in black, Fig. 3D), with a concomitant appearance of clusters of the *Nrf2*^*+/+*^ genotype (only representing 3.42%, as shown in Fig. 3D, in green) at 3 weeks (Fig. 3D). A notable feature is the behavior of clusters 1, 7, and 10, which represent alveolar type 2 cells (cluster 1), alveolar type 1 cells (cluster 7), and epithelial cells (clusters 10) as previously described in Fig. 2B–D. Consistent with the fact that the primary cell types of origin for malignant transformation is alveolar type II cells, there is an enrichment of alveolar type II cells (cluster 1, shown in blue) exclusively displayed in *Kras*^*G12D/+*^;*Nrf2*^*+/+*^cells in the control group. These clusters, including cluster 1, reached their peak proportions two weeks after TAM treatment in the *Kras*^*G12D/+*^;*Nrf2*^*−/−*^ background, coinciding with peak *Nrf2* deletion. However, by three weeks, these clusters exhibited a decline in the *Kras*^*G12D/+*^;*Nrf2*^*−/−*^ genotype, with some proportions reappeared and become enriched in *Kras*^*G12D/+*^;*Nrf2*^*+/+*^ populations (Fig. 3D). This reversal suggests that the loss of *Nrf2* in the *Kras*^*G12D/+*^ background compromises alveolar- and epithelial-derived cancer cell survival. Together, these results underscore the *Kras*-genotype-specific effects of *Nrf2* deletion, where *Nrf2* loss selectively disadvantages *Kras*^*G12D/+*^ cancer cells, supporting NRF2's critical role in tumor maintenance.

### 2.4 *Nrf2* deletion induces ferroptosis-related gene expression in *Kras*^*G12D*^ cancer cells

NRF2 is a critical regulator of intracellular iron homeostasis and lipid peroxidation, two fundamental processes that regulate ferroptosis. *Ptgs2*, *Acsl4*, and *Tfrc* are well-established ferroptosis-associated genes and are commonly used as transcriptional markers of ferroptotic responses. To further define the relationship between NRF2 loss and ferroptosis-associated transcriptional changes, we analyzed the expression of key ferroptosis-related genes (*Ptgs2*, *Acsl4*, and *Tfrc*) following TAM-induced *Nrf2* deletion. Heatmaps and UMAP visualizations (Fig. 4A and B) demonstrate distinct temporal and genotype-specific shifts in gene expression.

In *Kras*^*G12D/+*^;*Nrf2*^*−/−*^ cells, the mRNA levels of *Ptgs2*, *Acsl4*, and *Tfrc* increased in 1, 2, and 3 weeks post-TAM treatment compared to control (Fig. 4A, in black, right panel: intensity; left panel: % of cells expressing the indicated genes). In contrast, such temporal increases were absent in *Kras*^*+/+*^ genotype groups following *Nrf2* deletion. These results reinforce the link between *Nrf2* deletion and ferroptosis activation, suggesting that *Nrf2* deletion selectively sensitizes *Kras*^*G12D/+*^ cancer cells to ferroptosis.

UMAP analysis (Fig. 4B) further emphasizes the spatial clustering of ferroptosis-related gene expression. The mRNA levels of *Acsl4*, *Ptgs2* and *Tfrc* were predominantly upregulated in alveolar type II cells (Cluster 1, shown in blue in Fig. 2B). These genes reached their peak expression at two weeks post-TAM treatment and declined at three weeks. These results provide evidence that NRF2-mediated suppression of ferroptosis is critical for tumor cell survival in *Kras*^*G12D*^-driven lung cancers.

### 2.5 *Nrf2* deletion in *Kras*^*G12D*^ tumors promotes ferroptosis

To confirm the scRNA-seq data indicating that *Nrf2* deletion in cancer cells leads to increased ferroptotic cell death, we analyzed NRF2 expression and ferroptosis induction at different time points following TAM treatment using both immunoblot and immunofluorescence analyses (Fig. 5A and B). This experiment was performed using a separate 8-week endpoint cohort and therefore was not directly time matched to the scRNA-seq analysis shown in Fig. 2, Fig. 3, Fig. 4. Residual NRF2 signal in whole-lung lysates likely reflects the mixed cellular composition of bulk lung tissue and incomplete or asynchronous recombination across all lung cell populations.

Tumors at 8 weeks post-FlpO instillation (Group 2) exhibited the highest expression of NRF2 and its target genes, reflecting *Kras*^*G12D*^-driven NRF2 upregulation in cancer cells [19]. TAM treatment progressively reduced *Nrf2* expression, leading to a corresponding downregulation of its targets Akr1c6/18 and Slc7a11, as observed in the immunoblot analyses (Fig. 5A). Similar reductions were detected by immunofluorescence staining (Fig. 5B). As NRF2 and its target gene expression decreased, ferroptosis markers SO_2/3_-PRDX3, COX2, ACSL4, and TFR1 showed a progressive increase while PRDX3 remained unchanged (Fig. 5A and B).

To enable direct comparison of PRDX3 oxidation status, SO_2_/_3_-PRDX3 and total PRDX3 were analyzed on serial (adjacent) tissue sections from the same lung regions. SO_2_/_3_-PRDX3, which reflects acute mitochondrial oxidative stress, increased transiently following tamoxifen-induced *Nrf2* deletion and subsequently declined at later time points as NRF2-deficient tumor cells were eliminated. This biphasic behavior is consistent with the genotype-resolved scRNA-seq analysis (Fig. 3C), which shows an initial accumulation followed by selective loss of *Kras*^*G12D/+*^*;Nrf2*^*−/−*^ tumor cells over time. The moderate increase in ferroptosis markers is also consistent with the rapid elimination of ferroptotic cancer cells observed in the single-cell analyses shown in Fig. 3, Fig. 4.

Collectively, these findings demonstrate that *Nrf2* deletion suppresses its downstream targets while promoting ferroptosis, reinforcing NRF2's role in protecting *Kras*^*G12D*^-driven lung cancer cells from ferroptotic cell death. Additional immunofluorescence images of NRF2, its targets, and ferroptosis markers, together with DAPI nuclear staining and merged images, are shown in Supplementary Fig. S2A (NRF2 and its targets) and **Supplementary S2B** (ferroptosis markers)**, quantified in Supplementary S2C.**

### 2.6 *NRF2* deletion induces ferroptosis in isolated mouse *Kras*^*G12D*^-driven lung cancer cells

**Fig. 6:**
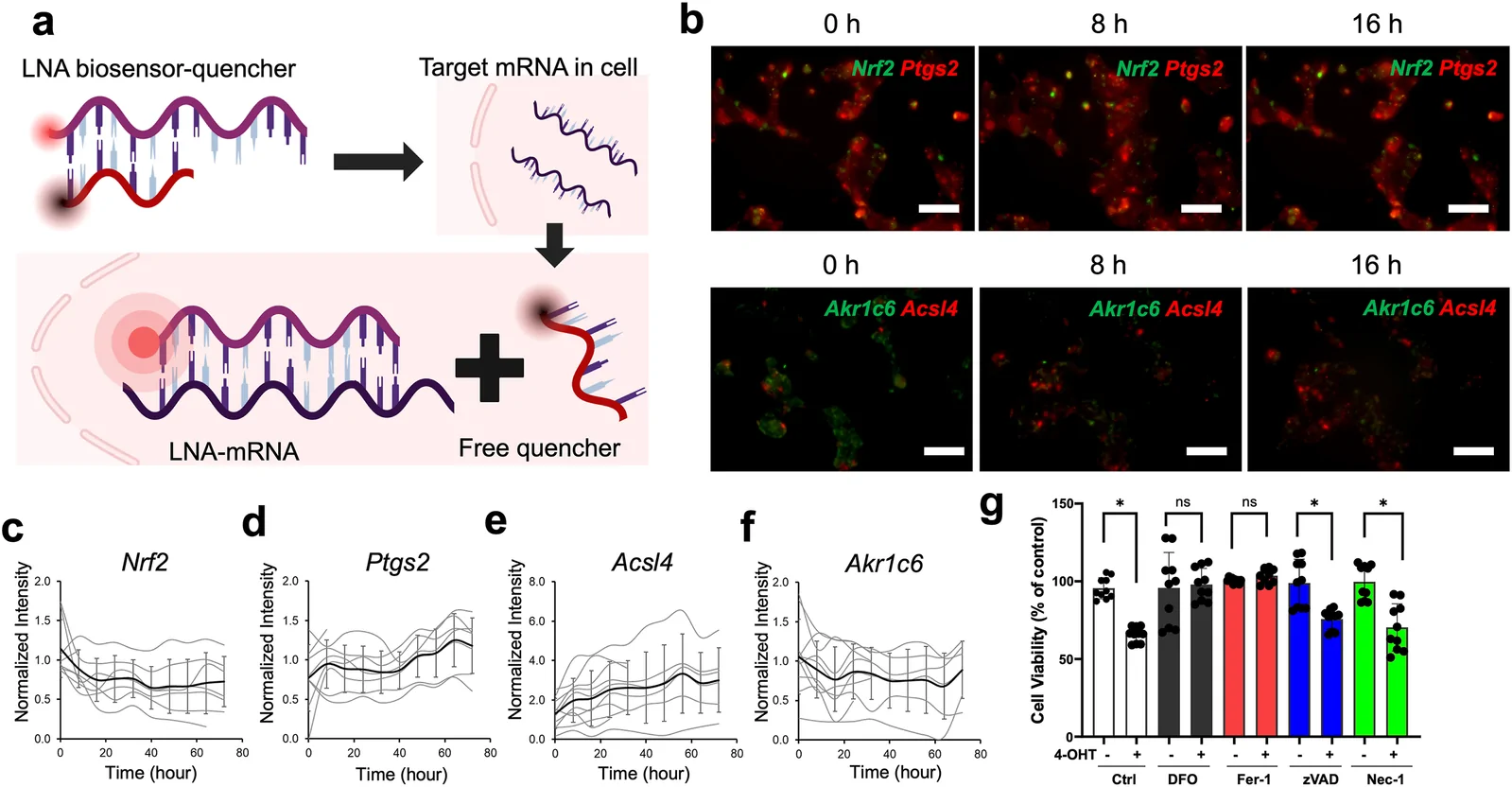
***NRF2 deletion induces ferroptosis in isolated mouse Kras*^*G12D*^*-driven lung cancer cells.*** a, Schematic representation of live single-cell biosensors designed to track mRNA expression in individual cells. The fluorophore-conjugated LNA probe is initially quenched by a quencher probe. Upon hybridization with the target mRNA, the quencher is displaced, enabling fluorescence-based real-time tracking of mRNA expression. **b,** Representative images of cells treated with 4-OHT (0 h corresponds to the time when culture dishes were placed on the microscope after 24 h of TAM treatment, marking the optical time point for *NRF2* deletion tracking). Scale bars, 100 μm. **c-f,** Tracking of *Nrf2*, *Ptgs2*, *Acsl4*, and *Akr1c6* mRNA in individual cells treated with 4-OHT. Data were normalized to the control to minimize the influence of photobleaching. Gray lines represent biosensor intensity traces for single cells, where shorter traces indicate cell death during the experiment. Black lines show the average expression of all analyzed cells. Data are presented as mean ± S.D. n = 8 for *Nrf2* and *Ptgs2*; *n* = 8 for *Acsl4* and *Akr1c6*. Images were acquired at 8-h intervals over a total recording period of 72 h. Representative time-lapse videos are provided as Supplementary Movies S1–S2. **g**, **Pharmacological inhibition of ferroptosis rescues *Nrf2*-deleted cancer cell death.** Quantification of cell survival in isolated KNR mouse lung cancer cells cultured in 3D, treated with 4-OHT for 72 h to induce Nrf2 deletion, followed by treatment with DMSO (Ctrl), 5 μM deferoxamine (DFO), 0.5 μM ferrostatin-1 (Fer-1), 50 μM zVAD-FMK (zVAD), or 50 μM necrostatin-1 (Nec-1) for 20 h. Cell survival was normalized to non-4-OHT-treated control group (set to 1.0). Data represent mean ± SD; n = 10 per condition. Statistical comparisons were performed between − 4-OHT and + 4-OHT for each treatment group. Statistical significance was determined using an unpaired two-tailed Student's t-test. *P < 0.05, ns = not significant.

We further investigated the effect of *Nrf2* deletion on ferroptosis at the mRNA level using time-lapse fluorescence microscopy and live single-cell biosensors [28,29]. Isolated KNR mouse lung cancer cells were transfected with a biosensor that reversibly hybridizes to intracellular mRNA of interest, activating fluorescence upon hybridization and allowing real-time tracking of mRNA expression in live cells (Fig. 6A and B). Four biosensor probe sequences were designed and used in this study (see Supplementary Methods). These probes were either previously validated [19] or confirmed through treatment with the ferroptosis inducer, imidazole ketone erastin (IKE) (Supplementary Fig. S3).

Upon 4-hydroxytamoxifen (4-OHT) treatment, a decrease in *Nrf2* mRNA was observed among most cells (Fig. 6C). Notably, Nrf2 mRNA levels decreased substantially after the 8 h imaging time point, resulting in a marked decline during the 16 h of observation (Fig. 6C). A high degree of heterogeneity was noted in both mRNA expression levels and their dynamics among individual cells. Despite this variability, an initial decrease in expression was observed, with average intensity levels stabilizing after approximately 20 h (equivalent to 2 days post-4-OHT treatment). In contrast, *Ptgs2* and *Acsl4* mRNA levels displayed an increased expression, as indicated by sensor intensity values continuing to rise over 72 h (4d post-OHT treatment), suggesting the accumulation of these ferroptosis markers (Fig. 6D and E). Similar to *Nrf2*, *Akr1c6* also exhibited an initial decline, stabilizing after approximately 20 h (Fig. 6F). These findings further support the notion that *Nrf2* deletion promotes ferroptosis in *Kras*^*G12D/+*^ cancer cells.

Consistent with the observed changes in mRNA levels, immunoblot and immunofluorescence analyses revealed a progressive decrease in NRF2 protein expression following 4-OHT treatment, accompanied by the downregulation of its target proteins, including AKR1C6/18 (*Akr1c6* and *Akr1c18*, murine orthologs of human *AKR1C1*), SLC7A11, ME1, AKR1B8 (*Akr1b8*, a murine ortholog of human *AKR1B10*), G6PD, and HO-1. In contrast, ferroptosis markers, SO_2/3_-PRDX3 (PRDX3 as a control), COX2, ACSL4, and 4-HNE were upregulated over time. (Supplementary Figs. S4-S5). These protein-level changes align with the single-cell mRNA dynamics, further reinforcing that *Nrf2* deletion sensitizes *Kras*^*G12D/+*^ cancer cells to ferroptosis.

To determine whether ferroptosis is the predominant form of cell death induced by NRF2 deletion, we treated isolated KNR mouse lung cancer cells cultured in 3D with ferroptosis (DFO, Fer-1), apoptosis (zVAD), or necroptosis (Nec-1) inhibitors following 4-OHT–induced Nrf2 deletion. As shown in Fig. 6G, DFO and ferrostatin-1 significantly rescued cell survival, whereas zVAD and Nec-1 had no effect, demonstrating that NRF2 deletion–induced tumor cell death is ferroptosis-dependent.

## 3 Discussion

Previously, we reported that NRF2 activation via sulforaphane prior to tumor induction prevented the initiation of lung tumors induced by chemical carcinogens but had no effect on *Kras*^*G12D*^-driven tumor initiation. However, NRF2 activation post-tumor initiation significantly accelerated the progression of pre-existing tumors in both models. Conversely, NRF2 inhibition with Brusatol prior to tumor induction increased tumor initiation in the chemical carcinogen-induced model but did not impact *Kras*^*G12D*^-driven tumorigenesis. In contrast, NRF2 inhibition after tumor initiation significantly slowed tumor progression in both models [30]. These findings highlight NRF2's dual, context-dependent roles in tumor biology: acting as a tumor suppressor during the pre-tumor initiation phase by mitigating cellular stress and damage to prevent malignant transformation yet functioning as a tumor promoter in established cancers by sustaining the survival of transformed cells. Consistent with this, a recent study demonstrated that NRF2 activation via *Keap1*^*R554Q*^ or *Nrf2*^*D29H*^ knock-in enhances tumor progression in *Kras*^*G12D*^-driven lung cancer models [31].

Despite three decades of dedicated research effort in the NRF2 field, the absence of NRF2-specific inhibitors has hindered efforts to fully elucidate the precise role and underlying mechanisms of NRF2 in tumor maintenance. We identified Brusatol using an ARE-luciferase cell-based screening assay and identified its role as a global protein synthesis inhibitor [32,33]. While Brusatol effectively reduces NRF2 protein levels due to NRF2's short half-life, a feature invaluable for studying NRF2's role in chemoresistance and tumorigenesis [19,30,32], its potential off-target effects limit its suitability for rigorous mechanistic investigations. This underscores the need for highly specific NRF2 inhibitors or genetic models that allow precise and complete NRF2 inhibition post-tumor initiation.

A key advance of this study is the resolution of NRF2's opposing roles in tumor initiation versus tumor progression through the use of an inducible genetic model that enables post-initiation *Nrf2* deletion *in vivo*. By enabling complete and temporally controlled *Nrf2* deletion after tumor formation, the KNR model reveals that established *KRAS*-driven tumors acquire a specific dependency on NRF2 for survival, a question that could not be addressed using whole-body *Nrf2*/*Keap1* mouse models.

To implement this approach, we employed KNR mice, an inducible *Nrf2* knockout model that permits precise, post-initiation deletion of *Nrf2* in tumor-bearing lungs. This model enabled direct interrogation of NRF2 function during tumor maintenance, independent of its roles in tumor initiation. Because tamoxifen-induced Cre activation deletes *Nrf2* in both *Kras*^*G12D*^ tumor cells and surrounding non-cancerous lung, stromal, and immune cells, we cannot completely exclude contributions from NRF2 loss in the tumor microenvironment to the *in vivo* phenotype. Using this system, we integrated scRNA-seq with AI-assisted neural network–based genotype classification, together with cellular, biochemical, and functional validation and real-time tracking of *Nrf2* loss and ferroptosis-associated responses in living cells. We acknowledge that genotype assignments inferred from scRNA-seq data are model-based predictions and not definitive per-cell genotype calls; therefore, these analyses were interpreted together with exon-specific read coverage, histological evidence of tumor regression, suppression of NRF2 target genes, induction of ferroptosis markers, and ex vivo validation in isolated KNR tumor cells. Together, these complementary approaches provide compelling evidence that NRF2 is indispensable for the survival of established tumors. Remarkably, inducing *Nrf2* deletion as late as 16 weeks after tumor initiation led to a striking survival benefit, significantly prolonging median survival from 86 days in control mice to 141 days in Nrf2-deleted mice (Fig. 1E). These findings establish NRF2 as a critical driver of tumor maintenance.

The mechanistic insights gained from this study reveal that cancer cells within the tumor microenvironment are continuously engaged in a battle against ferroptotic cell death to sustain their survival. This finding was facilitated by the recent identification of hyperoxidized PRDX3 as a specific ferroptosis marker [34,35]. Although our prior work demonstrated that *Nrf2* deletion sensitizes cancer cells to ferroptosis inducers more effectively than to other therapeutic agents [26], the current study uncovers a crucial and previously unappreciated insight: cancer cells rely on NRF2 to overcome intrinsic ferroptotic stress even in the absence of pharmacological intervention. Importantly, NRF2 was shown to be essential for the survival of cancer cells but dispensable for non-cancerous cells, reinforcing its function as a key suppressor of ferroptosis in the tumor context. Together, these findings establish, for the first time, that NRF2 is indispensable for tumor maintenance, independent of external therapeutic pressures.

Building on these findings, we propose that targeting NRF2 with inhibitors alone, without requiring combination therapies, could selectively eliminate cancer cells by exploiting their intrinsic ferroptotic vulnerability without harming non-cancerous cells. Our findings fundamentally redefine the NRF2-targeting paradigm, shifting it from a mediator of therapy resistance and a co-treatment target with chemotherapeutic agents to a standalone, transformative cancer therapy. Because loss-of-function *KEAP1*-mutant and gain-of-function *NRF2*-mutant tumors exhibit constitutive NRF2 activation, they may be particularly dependent on NRF2-mediated survival pathways and therefore especially susceptible to therapeutic NRF2 inhibition. Although the present study was performed in a Kras-driven lung tumor model, future studies will be needed to determine whether NRF2-dependent suppression of endogenous ferroptotic stress represents a broader tumor maintenance mechanism across additional cancer types and oncogenic drivers. Future studies should (1) validate NRF2's role in tumor maintenance across additional cancer types, and (2) accelerate the development of highly potent, selective NRF2 inhibitors. Such inhibitors could revolutionize cancer therapy by disrupting a fundamental cancer cell survival mechanism, paving the way for curative treatments against multiple malignancies.

## 4 Materials and methods

Chemicals, reagents, common methods, and detailed protocols are provided in the Supplementary Information (SI). All cell lines were authenticated by STR profiling (UF Scripps Core Facility) and tested negative for Mycoplasma contamination before use. Data are presented as mean ± SD, and n values represent biological replicates. Statistical analyses were performed using GraphPad Prism v9.0 (RRID:SCR_002798).

### 4.1 Mouse lung cancer models

Generation of *Kras*^*FSF.G12D/+*^;*Nrf2*^*Fl/Fl*^;*Rosa26*^*CreERT2/CreERT2*^ (KNR) mice. KNR mice were generated through a series of genetic crossings. Initial cross: *Nrf2*^*Fl/Fl*^ × *Rosa26*^*CreERT2/+*^ → *Nrf2*^*Fl/+*^; *Rosa26*^*CreERT2/+*^. Second cross: *Kras*^*FSF.G12D/+*^ × *Nrf2*^*Fl/+*^; *Rosa26*^*CreERT2/+*^ → *Kras*^*FSF.G12D/+*^; *Nrf2*^*Fl/+*^; *Rosa26*^*CreERT2/+*^. Final Cross: *Kras*^*FSF.G12D/+*^; *Nrf2*^*Fl/+*^; *Rosa26*^*CreERT2/+*^ × *Kras*^*FSF.G12D/+*^; *Nrf2*^*Fl/+*^; *Rosa26*^*CreERT2/+*^→ *Kras*^*FSF.G12D/+*^; *Nrf2*^*Fl/Fl*^; *Rosa26*^*CreERT2/CreERT2*^ (KNR).

All mice were maintained under specific pathogen–free conditions, and both sexes (8–10 weeks old, C57BL/6 background) were used in all experiments. All procedures were approved by the Institutional Animal Care and Use Committee at the University of Florida, Jupiter (IACUC protocol no. 24-002-01, Animal Welfare Assurance no. D16-00726). Mice were randomly assigned to experimental groups, and investigators were blinded to genotype and treatment during data collection and analysis.

### 4.2 Lung Tumor initiation and *Nrf2* deletion in KNR mice

*Tumor initiation:* Eight-week-old KNR mice were instilled intratracheally with FlpO virus (2.75 × 10^8^ pfu; University of Iowa Viral Vector Core Facility) to initiate tumor formation or with GFP virus (University of Iowa Viral Vector Core Facility) as a control.

*Nrf2 deletion:* KNR mice were administered tamoxifen (Sigma-Aldrich, Cat. no. T5648, CAS:10540-29-1) intraperitoneally (100 mg/kg body weight, daily for five consecutive days). The tamoxifen regimen was identical across all *in vivo* experiments. For tumor burden studies, tamoxifen was administered at the indicated times after FlpO instillation and lungs were harvested 8 weeks after tumor initiation. For survival studies, tamoxifen was administered 16 weeks after FlpO instillation. For scRNA-seq studies, lungs were harvested 22 weeks after FlpO instillation to obtain sufficient tumor cells for single-cell RNA-seq analysis, and tamoxifen was administered 1, 2, or 3 weeks before harvest.

### 4.3 Single-cell RNA sequencing and neural network-based genotype classification

Single-cell RNA sequencing (scRNA-seq) libraries were generated, sequenced, and mapped to the mouse reference genome (GRCm38/mm10; UCSC) using the 10x Genomics Cell Ranger pipeline (v7.1.0, RRID:SCR_017344). Data processing was performed in R (v4.3.1, RRID:SCR_001905) using the Seurat package (v4.4.0, RRID:SCR_007322) [36], and batch effects were corrected by integrating all samples before downstream analysis. Cell clustering was conducted using Seurat's FindClusters function, and cluster identities were assigned based on marker gene expression.

To classify cells by genotype, binary classification neural network models were developed using PyTorch (v2.0, RRID:SCR_018536) and trained on an NVIDIA A100 GPU (RRID:SCR_021876). Two independent models were trained: one to distinguish *Kras*^*+/+*^ from *Kras*^*G12D/+*^ and another to differentiate *Nrf2*^*+/+*^ from *Nrf2*^*−/−*^. Both models shared an architecture of three layers (width: 30), with a dropout rate of 0.1 and ReLU activation.

The labeled datasets consisted of single-cell transcriptomes with at least 10 mapped reads to either the *Kras*^*G12*^ locus (chr6:145,246,771; mm10 reference) or the *Nrf2* exon 4–5 splice junction. *Kras*^*G12D*^ mutant cells were identified as those with >50% of reads at this locus exhibiting a C-to-T transition. *Nrf2* knockout cells were defined by complete skipping of exon 5.

Gene expression input features consisted of raw read counts for 32,285 genes per cell. To reduce input dimensionality, genes with zero counts across all cells were removed: 13,641 genes from the *Kras*-labeled dataset and 6882 genes from the *Nrf2*-labeled dataset. The *Kras*-labeled dataset comprised 211 cells, evenly split between *Kras*^*+/+*^ and *Kras*^*G12D/+*^ genotypes. The *Nrf2*-labeled dataset included 8706 cells, also evenly split between *Nrf2*^*+/+*^ and *Nrf2*^*−/−*^ genotypes.

Due to the small size of the *Kras*-labeled dataset, it was first partitioned into training/validation (80%) and a held-out test set (20%) for k-fold cross-validation. This allowed for a better estimate for model performance while hyperparameter tuning. For the final models, both the *Kras* and *Nrf2*-labeled datasets were randomly split into training (80%), validation (10%) and test (10%) with no overlap.

To assess reproducibility and mitigate classification variance due to limited training data, two independent model sets were trained for each genotype. The average performance of the two *Kras* models was 98.3% on the training set, 96.1% on the validation set, and 86.4% on the test set. For the Nrf2 models, average performance was 95.5% (training), 89.4% (validation), and 88.3% (test). For the final classification, one *Kras* model was paired with one *Nrf2* model to assign each cell to one of four classes. Only cells that were consistently classified across both paired models were used for downstream analyses of temporal shifts in cell populations following *Nrf2* deletion.

Model-based genotype predictions were used to stratify scRNA-seq cell populations for exploratory genotype-resolved analysis. Because these assignments are inferential and based on sparse single-cell transcriptomic reads, they were not treated as definitive per-cell genotype calls and were interpreted together with exon-specific read coverage and independent histological, molecular, and functional validation.

### 4.4 LNA biosensors for tracking mRNA in live single cells

Lock nucleic acid (LNA) biosensors were designed to track mRNA expression in live cells by retrieving *Nrf2, Ptgs2, Acsl4,* and *Akr1c6* sequences following established protocols [28,29]. The LNA probes were synthesized with fluorescent labels and paired with quencher sequences to enable real-time detection of mRNA expression upon target recognition. For live single-cell tracking, the probes were transfected using Lipofectamine 3000 (Thermo Fisher, Cat. no. L3000-015, RRID:SCR_015690), and fluorescence signals were imaged using a ZEISS LSM 980 confocal microscope (RRID:SCR_020925). Images were acquired at 8-h intervals over a total recording period of 72 h. This interval was chosen to minimize photobleaching.

## CRediT authorship contribution statement

**Dichun Huang:** Data curation, Formal analysis, Investigation. **Mae Zhang:** Data curation, Formal analysis. **Ben N. Stansfield:** Data curation, Formal analysis. **Mona Ahmed:** Data curation. **Tianbao Xu:** Data curation. **Lewis Alexander:** Data curation. **Annadurai Anandhan:** Data curation. **Mengjiao Ma:** Data curation. **Pengfei Liu:** Data curation. **Yang Yang-Hartwich:** Methodology. **Deyu Fang:** Supervision. **Eli Chapman:** Supervision. **Joe G.N. Garcia:** Supervision. **Pak Kin Wong:** Supervision. **Aikseng Ooi:** Supervision. **Donna D. Zhang:** Writing – review & editing.

## Ethics declaration

This study was conducted in accordance with the following guidelines for animal welfare and/or reporting: NIH Principles and Guidelines for Reporting Preclinical Research. This study was approved by the IACUC.

## Declaration of competing interests

The authors declare no competing financial or non-financial interests related to the content of this article.

## Data Availability

The raw scRNA-seq data have been deposited in the Sequence Read Archive (SRA) and are accessible under accession number PRJNA1208450. Source code and scripts are available at https://github.com/OoiLab-at-UF/NRF2-Mediated-Ferroptosis-Suppression-Defines-a-Cancer-Specific-Vulnerability-in-Tumors.git. Training datasets are available at https://huggingface.co/datasets/aikseng/Kras-Nrf2-Dataset.
