# Supplementary material for: NRF2-mediated ferroptosis suppression defines a cancer-specific vulnerability in tumors
Source: Redox Biol. 2026 Aug 19;96:104352. doi: 10.1016/j.redox.2026.104352 (PMC13524513; doi:10.1016/j.redox.2026.104352)
Supplement: Supplementary file 1 [file mmc1.docx]

**Supplementary Information**

**NRF2-Mediated Ferroptosis Suppression Defines a Cancer-Specific Vulnerability in Tumors**

This file includes: Supplementary Figures S1–S5, SI Methods, and Supplementary References.

**Section 1: Supplementary Figures S1-S5**

**Fig. S1.**

**Fig. S1. *NRF2* KO cell lines were unable to develop into sizable tumors in NSG mice.** **a,** Sets of A549-*NRF2^+/+^* (WT), A549-*NRF2^+/-^* (Het-4), and A549-*NRF2^-/-^* clones (KO-2, and KO-6); and **b**, SKOV-3-*NRF2^+/+^* (WT), SKOV-3-*NRF2^+/-^* (Het), and SKOV-3-*NRF2^-/-^* (KO-1, KO-2, KO-3) were subcutaneously injected into NSG mice to assess tumor growth. Tumor volumes were measured and presented as the mean tumor burden (mm^3^) over time. Data represent mean ± SEM. n = 10 per group.

**Fig. S2a**

**Supplementary Fig. S2b**

**Supplementary Fig. S2c**

**Supplementary Fig. S2 (related to Fig 5b). Immunofluorescence analysis of NRF2, its downstream targets, and ferroptosis markers in *Kras^G12D^*-driven lung tumors following TAM treatment.**

Representative immunofluorescence images of **a**, NRF2 and its downstream targets, AKR1C6/18, SLC7A11, NQO1, ME1, AKR1B8, G6PD, HO-1, and γ-GCS; **b,** ferroptosis markers, SO_2/3_-PRDX3 (PRDX3 as a control), COX2, ACSL4, TfR1, and 4-HNE in lung tumor tissues from Groups 1–5 at 8 weeks after FlpO instillation. DAPI staining identifies nuclei, and merged images show the overlay of DAPI with the signal from the indicated proteins. Group 1-5 described in Figure 1: no tumor (group 1), no-TAM (group2), TAM treatment for 2 weeks (group 3), 4 weeks (group 4), or 6 weeks (group 5). Scale bar, 50 μm. **c,** Quantification of immunofluorescence signal intensity from panels a and b. Fluorescence intensities of the indicated NRF2 target proteins and ferroptosis-associated markers was quantified from representative images and normalized to the corresponding control group (Group 2). Data are presented as mean ± SD (n = 3 biological replicates). Statistical significance was determined by one-way ANOVA followed by Tukey’s multiple-comparison test. *P < 0.05, **P < 0.01; ns, not significant.

**Supplementary Fig. S3**

**Supplementary Fig. S3. Induction of *Ptgs2 and Acsl4* mRNA by IKE. A-B,** Tracking of *Ptgs2* and *Acsl4* mRNA in individual cells treated with IKE. An increasing trend was observed for both *Ptgs2* and *Acsl4* following IKE treatment. Data were normalized to the control to minimize the influence of photobleaching. Gray lines represent biosensor intensity traces for single cells, where shorter traces indicate cell death during the experiment. Black lines show the average expression of all analyzed cells. Data are presented as mean ± S.D. n = 8 for *Ptgs2*, and n = 6 for *Acsl4*. Imaging was performed at 8-hour intervals for a duration of 72 hours.

**Supplementary Fig. S4**

**Supplementary Fig. S4. *Nrf2* deletion induces ferroptosis in mouse *Kras^G12D^*-driven lung cancer cells. a,** Immunoblot analysis shows the expression of NRF2, its target genes (AKR1C6/18, SLC7A11, ME1, AKR1B8, G6PD, and HO-1), and ferroptosis-associated markers (SO_2/3_-PRDX3 vs. PRDX3, COX2, and ACSL4). GAPDH was used as a loading control.. Protein expression was normalized to GAPDH and quantified by densitometry. **b**, Immunofluorescence analysis of KNR mouse lung cancer cells treated with 4-hydroxytamoxifen (4-OHT, 2 μM) for up to five days. The temporal expression patterns of NRF2, AKR1C6/18, SLC7A11, SO_2/3_-PRDX3, and PRDX3 were assessed after 4-OHT treatment. Scale bar, 50 μm.

**Supplementary Fig. S5**

 **Fig. S5. Immunofluorescence analysis of NRF2, its targets, and ferroptosis markers in mouse *Kras^G12D^*-driven lung cancer cells.**

Representative immunofluorescence images of NRF2, and its downstream targets, (AKR1C6/18, SLC7A11, NQO1, ME1, AKR1B8, G6PD, HO-1) and ferroptosis markers (SO_2/3_-PRDX3 vs PRDX3, COX2, ACSL4, and 4-HNE) in mouse *Kras^G12D^*-driven lung cancer cells treated with 4-OHT (2 μM) for up to five days. DAPI staining identifies nuclei, and merged images show the overlay of DAPI with the signal from the indicated proteins. Scale bar, 50 μm. Fluorescence intensities of the indicated NRF2 target proteins and ferroptosis-associated markers were quantified from representative images and normalized to the day 0 control. Data are presented as mean ± SD (n = 3 biological replicates). Statistical significance was determined by one-way ANOVA followed by Tukey’s multiple-comparison test. *P < 0.05, **P < 0.01; ns, not significant.

**Section 2: SI Materials and Methods**

**Chemicals and Reagents**

Imidazole ketone erastin (IKE) was a gift from Dr. Brent Stockwell. FlpO virus and the control GFP virus were purchased from the University of Iowa Viral Vector Core Facility. Tamoxifen (T5648) and 4-hydroxytamoxifen (H7904) were purchased from Sigma-Aldrich.

The Antibodies used in this study are listed in the table below:

| **Antibody** | **SOURCE** | **IDENTIFIER** |
| --- | --- | --- |
| ACSL4/FACL4 | Proteintech | Cat. no. 22401-1-AP |
| PRDX3-SO_2/3_ | Dr. Jin Ye ‘s Laboratory | N/A |
| Peroxiredoxin 3/PRDX3 | Abcam | Cat. no. ab128953 |
| Heme Oxygenase 1/HMOX1 (F-4) | Santa Cruz Biotechnology | Cat. no-390991 |
| γ-GCSm (E-4) | Santa Cruz Biotechnology | Cat. no. sc-55586 |
| AKR1B10 (AA-1) | Santa Cruz Biotechnology | Cat. no. sc-100501 |
| NQO1 (A180) | Santa Cruz Biotechnology | Cat. no. sc-32793 |
| CD71/TFRC/Transferrin Receptor (3B8 2A1) | Santa Cruz Biotechnology | Cat. no. sc-32272 |
| AKR1C1  DD (C-12) | Santa Cruz Biotechnology | Cat. no. sc-166297 |
| Malic Enzyme1  ME1 (C-6) | Santa Cruz Biotechnology | Cat. no. sc-365891 |
| NRF2 (H-300) | Santa Cruz Biotechnology | Cat. no. sc-13032  (discontinued) |
| COX2 | Cell Signaling Technology | Cat. no. 4842S |
| G6PD | Cell Signaling Technology | Cat. no. 8866S |
| SLC7A11/xCT a | Proteintech | Cat. no. 26864-1-AP |
| 4-Hydroxynonenal | Abcam | Cat. no. ab46545 |
| GAPDH (6C5) | Santa Cruz Biotechnology | Cat. no. sc-32233 |
| Beta Actin | Proteintech | Cat. no. 60008-1-Ig |
| Alpha Tubulin | Proteintech | Cat. no. 11224-1-AP |
| Goat anti-Mouse IgG (H+L), Alexa Fluor™ 488 | Thermo Fisher Scientific | Cat. no. A-11029 |
| Goat anti-Rabbit IgG (H+L), Alexa Fluor™ 594 | Thermo Fisher Scientific | Cat. no. A-11037 |

**Immunoblot Analysis (IB)**

Cells and tissue lysates were harvested in sample buffer, boiled, sonicated, and resolved by SDS-PAGE. Proteins were transferred to membranes and incubated with primary antibodies for 1 h, washed, and incubated with HRP-conjugated secondary antibodies for 1 h.

**Immunofluorescence (IF)**

For cell-based immunofluorescence, cells growing on glass coverslips were fixed with 100% methanol (-20°C for 10 min). For tissue-based immunofluorescence, samples were embedded in OCT compound (Sakura 25608-930), rapidly frozen, and sectioned using a cryostat (Leica CM1950), then fixed in 4% paraformaldehyde for 10 min.

Fixed samples were then washed in PBS and blocked with 10% fetal bovine serum (FBS, GenClone 25-550) for 1 h at room temperature (RT). Samples were incubated with primary antibodies targeting relevant proteins for 1 h at RT, washed in PBS, and then incubated with fluorophore-conjugated secondary antibodies for 1 h at RT. After a final PBS wash, sections were mounted with antifade reagent. Fluorescent images were acquired using a Zeiss Observer.Z1 microscope and a confocal microscope (Olympus FV3000), and analyzed with ImageJ software.

**Hematoxylin and Eosin (H&E) and Immunohistochemistry (IHC) Staining**

Tissues were fixed in 10% neutral-buffered formalin (NBF) for 24–48 h, dehydrated through graded ethanol, cleared in xylene, and embedded in paraffin. Paraffin-embedded tissues were sectioned at 4–5 µm thickness and mounted on glass slides.

For H&E staining, sections were deparaffinized, rehydrated, and stained with hematoxylin (1–2 min), followed by eosin (30 sec–1 min). After dehydration through graded alcohol and clearing in xylene, slides were mounted with a permanent medium.

For IHC, antigen retrieval was performed using sodium citrate buffer (0.01 M, pH 6.0) at high temperature, followed by blocking of endogenous peroxidase activity with 0.3% H₂O₂. Sections were incubated with primary antibodies overnight at 4°C, and staining was performed using the EnVision+ System-HRP kit. Images were captured using a Nikon Eclipse 50i microscope, analyzed with NIS Elements software, and quantified using ImageJ.

**Cell culture and generation of *NRF2^−/−^ and NRF2^+/−^ cell lines***

A549 and SKOV-3 were purchased from ATCC. Cells were grown in DMEM (Gibco 11965-092) supplemented with GlutaMAX (Gibco 35050-061), 10% FBS, and 1% penicillin/streptomycin (Gibco 105070-063).

CRISPR knockout cell lines were generated using CRISPR-Cas9–mediated gene editing. We utilized sgRNA species previously designed and published by Wang and co-workers as an activity optimized pooled sgRNA library (1). In this pooled library, there are 10 different species of sgRNAs targeting each gene. For each gene, all 10 sgRNA species were cloned into the pSpCas9(BB)-GFP plasmid (2), and genome targeting efficiency of each sgRNA construct was determined by T7 endonuclease I assay. The highest efficiency sgRNAs were selected for making knockout cell lines. A549 and SKOV-3 cells were transfected with the selected sgRNA plasmid for 24 h, and GFP-positive cells were isolated using FACS and subsequently plated at low confluency. Once individual colonies were visible to the naked eye, they were isolated using a clonal ring and expanded individually. Successful homozygous/heterozygous knockout/knockdown of *NRF2* gene was confirmed by Sanger sequencing the sgRNA binding region (around 500 bp flanking the sgRNA binding region), and by immunoblot analysis.

The best sgRNAs used for making the *NRF2^−/−^* and *NRF2^+/−^* cell lines are listed below:

*NRF2*

sgRNA-1 5′-TATTTGACTTCAGTCAGCGA-3′

sgRNA-2 5′-TAGTTGTAACTGAGCGAAAA-3′

**Xenograft models**

NSG ([NOD.Cg-](https://www.jax.org/strain/005557)*[Prkdc](https://www.jax.org/strain/005557)^[scid](https://www.jax.org/strain/005557)^ [Il2rg](https://www.jax.org/strain/005557)^[tm1Wjl](https://www.jax.org/strain/005557)^*[/SzJ](https://www.jax.org/strain/005557)) female mice, 7-8 weeks old, were randomly allocated into groups and injected subcutaneously with 2 x 10^7^ A549-based or SKOV-3-based *NRF2* WT, Het or KO cells. Tumor volume was measured 3 times per week with an electronic caliper and calculated with the formula: 0.5 × length × width^2^. Mice were euthanized, and tumor volume data were analyzed in GraphPad Prism. Data are presented as mean ± SD, with significance determined by one-way ANOVA.

**Mouse lung cancer models**

All mice were housed in a specific pathogen-free room with ad libitum access to food and water at the UF-Scripps Animal Care facility. All protocols were approved by the UF Institutional Animal Care and Use Committee, and experiments were conducted in accordance with the Guide for the Care and Use of Laboratory Animals. *Kras^FSF.G12D/+^*, *Nrf2^Fl/+^*, and *Rosa26^CreERT2/+^* were originally purchased from the Jackson Laboratory and bred as described in the manuscript to generate *Kras^FSF.G12D/+^*; *Nrf2^Fl/Fl^*; *Rosa26^CreERT2/CreERT2^* (KNR) mice. The KNR mice were maintained and used for the experiments described in the manuscript.

At euthanasia, lungs were collected for gross tumor analysis and weight measurement. The lungs were then divided, with portions fixed in neutral-buffered formalin for histological analyses (H&E, IHC), embedded in OCT and rapidly frozen for IF, or snap-frozen for IB and RNA analyses.

**Single cell preparation**

The single cell suspension isolated from mouse lung was performed as described (3). Briefly, freshly isolated lung tissue (around 100 mg) was washed in cold PBS, chopped on a clean petri dish into fragments under ~0.4 mm and transferred into a 15-ml conical tube containing HBSS supplemented with 1,250 μg/ml pronase (Sigma Aldrich, 10165921001), 9.2 μg/ml elastase (Worthington, cat. no. LS006365) 100 μg/ml DNase I (Sigma Aldrich, 11284932001), 100 μg/ml Dispase (Sigma Aldrich, 4942078001), 1,500 μg/ml collagenase A (Sigma Aldrich, 10103578001) and 100 μg/ml collagenase IV (Thermo Fisher Scientific, NC9836075). Sample tissues were then incubated at 37°C with rotation for 10 min, followed by manual pipette dissociation and a further 10 min incubation at 37°C with rotation. The cell suspension was passed through a 70-μm cell strainer and centrifuged at 500 *g,* 4°C for 7 min. The supernatant was removed, and the pellet was resuspended in 400 μl of ACK RBC lysis buffer (Thermo Fisher Scientific, A1049201). The sample was incubated in ACK RBC lysis buffer for 1 min on ice, followed by the addition of ice cold PBS (2:1 v:v). The cells were pelleted by a short centrifugation for 8 s at 4°C using the short spin setting with centrifugal force ramping up to, but not exceeding, 11,000 *g*. The supernatant was removed. The pellet color was assessed; if RBCs remained (pellet color pink or red), the ACK step was repeated up to two additional times. The cells were pelleted using short centrifugation as described above. The pellet was resuspended in PBS, passed through a 70-μm cell strainer and the collected cells were centrifuged at 500 *g* for 7 min at 4°C.

**Fast depletion of CD45+ immune cells**

Following dissociation of lung tissue samples, depletion of CD45+ cells was performed using CD45 MicroBeads (Miltenyi Biotec, 130-045-801) as previously described (3). Briefly, the single-cell suspension was centrifuged at 500 *g* for 4 min at 4°C. The supernatant was removed, and the pellet was resuspended in 80 μl of MACS buffer per 10^6^ cells (PBS supplemented with 0.5% BSA, and 2 mM EDTA). MACS CD45 microbeads were added to the cell suspension (20 μl per 10^6^ cells). The cells were incubated on ice for 15 min. During incubation, the column was prepared by attaching the column to a MidiMACS separator and rinsing the column with 3 ml MACS buffer. Following incubation, the cells and bead conjugate were washed with 900 μl MACS buffer per 10^6^ cells. The cells were centrifuged at 500 *g* for 4 min at 4°C. The supernatant was removed, and the pellet was resuspended in 500 μl MACS buffer. The cell suspension was then transferred to the column and the effluent was collected (CD45^−^ fraction). The column was washed three times with 3 ml MACS buffer. The CD45^−^ fraction was centrifuged at 500 *g* for 4 min at 4°C. The pellet was resuspended in 50 μl of 0.4% BSA in PBS. Cells were counted and the concentration was adjusted 1,000 cells per μl.

**Library preparation, and Sequencing**

Single cell suspensions were processed for 3’ single cell RNA sequencing, using the 10xGenomics Chromium Single Cell 3’ Reagent Kit v3.1. 3’, was conducted by the University of Arizona Genetics Core. scRNAseq libraries were built according to the 10xGenomics protocol and were quantified by KAPA Illumina Library Quantification Kit. Libraries were sequenced by the UCLA Technology Center for Genomics & Bioinformatics on 1 lane of Illumina 2x100 NovaSeq S4.

**Binary Classification Neural Network Model Training (Detail)**

**Annotating dataset**

Note that training data came from all four groups (control, 1 week post-tamoxifen treatment, 2 weeks post-tamoxifen treatment, and 3 weeks post-tamoxifen treatment).

Samtools was used to extract mapped reads from BAM files generated by CellRanger. The mutation in *Kras* occurs at chr6:145,246,771 (UCSC mm10 reference genome), which encodes amino acid G12. A C-to-T transition results in an amino acid change from glycine (G) to aspartic acid (D), a known missense mutation that converts *Kras* from a proto-oncogene to an oncogene. Cells with approximately 50% of mapped reads at this region carrying a C-to-T transition were included in our training data as *Kras^G12D^* mutants. Conversely, cells with 100% of mapped reads at this region as C were included in our training data as *Kras* wild-type. Using this method, we obtained 211 balanced samples of wild-type and knockout genotypes for training and validation. Similarly, *Nrf2* knockout cells were defined as those where 100% of mapped reads skipped exon 5.

**Neural Network Architecture**

We trained a fully connected feedforward neural network to classify high-dimensional gene expression data. The architecture consisted of three hidden layers with 30, 40, and 30 neurons, respectively. Each hidden layer was followed by a LeakyReLU activation function and dropout (rate = 0.1) to mitigate overfitting.

Models were implemented in PyTorch using the torch.nn.Sequential module. The network architecture consisted of an input layer with dimensionality equal to the number of gene expression features, connected to a fully connected layer with 30 units, followed by a LeakyReLU activation function and a 10% dropout layer. This was followed by a second dense layer containing 40 units with LeakyReLU activation and dropout, and a third dense layer with 30 units, again followed by LeakyReLU activation and dropout. The final output layer contained two units for binary classification.

This architecture was designed to compress the input dimensionality and prevent overfitting through dropout regularization. LeakyReLU activations were used in place of standard ReLU to mitigate neuron inactivation due to vanishing gradients.

*Nrf2*-labeled and *Kras*-labeled datasets were split into training (80%), validation (10%), and test (10%) sets with no overlap. Due to the small size of the *Kras* dataset, k-fold cross validation was used to tune hyperparameters for the *Kras* models with training/validation (80%) and held out test set (20%). Model performance was assessed using accuracy on the test sets. Two independent models were trained for each classification task to evaluate reproducibility and reduce stochastic variability.

**scRNA-Sequencing data preprocessing**

Demultiplexing of sequencing data was performed with Illumina Bcl2fastq (v2.19.1.403) and reads were aligned to the mouse reference (GRCm38/mm10; UCSC) using the 10x Genomics CellRanger pipeline (v.7.1.0) achieving a total number of reads per sample of 219824474 ± 21008823 and an average number of reads per cell of 13793 ± 1972. Counts matrices, cell barcodes and cell features were exported and analyzed in R (v.4.3.1) using the Seurat package (v4.4.0) (4). This resulted in an scRNA-seq dataset containing 64568 cells across all samples. Cells were individually assigned to their corresponding genotypes *(Kras^+/+^*;*Nrf2^+/+^*, *Kras^+/+^*;*Nrf2^-/-^*, *Kras^G12D/+^*;*Nrf2^+/+^*, *Kras^G12D/+^*;*Nrf2^-/-^*) using our binary classification neural network models (Supplementary Table 1). Cells that were classified to more than one genotype were discarded from further analysis. A total of 50257 cells were then processed for further QC and analysis. Raw count was normalized and variance stabilized using the scTransform function in Seurat. Variable features (selection.method = "vst", nfeatures = 5000) were identified from 1 week post-*Nrf2* deletion and assigned as the anchor features that were used to integrate data and harmonize batch effects using the built in PrepSCTinegration, FindIntergrationAnchors and Intergrate functions in Seurat. The '1-week post-*Nrf2* deletion' sample was used to determine anchor features because it had the highest representation of all four genotypes.

**scRNA-Sequencing dimensionality reduction, clustering, and cell population assignment**

Integrated data underwent principal component analysis (npcs = 50), and a k-nearest neighbor graph was generated on the first 20 principal components, followed by shared nearest-neighbor (SNN) refinement to improve clustering resolution. Cell clustering was performed using the FindClusters function with a resolution of 0.5 prior to Uniform Manifold Approximation and Projection (UMAP) with the first 20 principal components. To assign cell populations, we curated a set of canonical marker genes corresponding to distinct cell types in the dataset. Cell populations were assigned based on the expression of the following markers: Endothelial cells (*Pecam1*, *Tek*) (5), alveolar type 1 cells (*Pdpn*, *Aqp5*) (6,7), alveolar type 2 cells (*Sftpc*) (8), epithelial cells (*Epcam*, *Cdh1*) (9), club cells (*Scgb3a1*, *Scgb3a2*, *Scgb1a1*) (10), mesothelial cells (*Msln*) (11), mesenchymal cells (*Pdgfra*, *Tcf21*, *Cdh11*) (12), Aerocytes (*Car4*) (13), alveolar fibroblasts (*Npnt*, *Ces1*, *Pdgfra*, *Plin2*) (14), adventitial fibroblasts (*Pi16*, *Dcn*) (14), amooth muscle cells (*Myh11*, *Myl9*, *Acta2*) (15-17), lymphatic endothelial cells (*Prox1*) (18), and pericytes (*Ebf1*, *Pdgfrb*, *Ng2*) (19,20).

**Biosensor design for tracking mRNA expression in live cells**

The mRNA sequences of *Nrf2, Ptgs2,* *Acsl4, and Akr1c6* were retrieved from the NCBI GenBank database. Secondary structure predictions of these sequences were generated using the UNAfold web server to identify accessible regions suitable for probe design. Candidate probe sequences were selected from these accessible regions and further screened using the NCBI Basic Local Alignment Search Tool for nucleotides (BLASTn) to ensure specificity to the target genes. For *Nrf2*, exon 5 of the mRNA was specifically targeted. The Integrated DNA Technologies (IDT) oligo tool was used to identify sequences with a GC content of 50–60% while excluding those prone to forming hairpin structures. The final probe sequences were synthesized by IDT. Each probe consists of 20 nucleotides arranged in an alternating pattern of DNA and locked nucleic acids (LNAs) and is fluorescently labeled at the 5′ end. To suppress fluorescence in the absence of the target, a 10-nucleotide complementary DNA quencher labeled with a quencher dye was designed to hybridize with each probe. Upon target recognition, the probe dissociates from the quencher, generating a detectable fluorescence signal.

Biosensor probe sequences

| *Nrf2* probe | Alex647N+TA+TC+TC+GG+AT+CA+AT+GC+GA+GC |
| --- | --- |
| *Nrf2* quencher | GATCCGAGATA3IAbRQSp |
| *Ptgs2* probe | Alex488N+CT+TC+AA+AT+GA+TG+TG+TA+CG+GC |
| *Ptgs2* quencher | TCATTTGAAG3BHQ_1 |
| *Acsl4* probe | Alex488N/+AG+CT+CG+GT+GG+TG+TG+GC+TG+AG |
| *Acsl4* quencher | CCACCGAGCT3BHQ_1 |
| *Akr1c6* probe | Alex647N+TG+GT+CC+TT+AA+AG+CT+AG+AA+CC |
| *Akr1c6* quencher | TTAAGGACCA3IAbRQSp |

+ indicates LNA monomers

**Live single cell tracking**

To perform the live single-cell tracking experiment, the quencher probe and fluorophore probes were first denatured at 95°C for 5 min, followed by gradual cooling to room temperature. The probe-quencher complexes were then incubated with Lipofectamine 3000 (Thermo Fisher Scientific, USA) for 10 min, following the manufacturer’s protocol. The resulting mixture was added to the cells dropwise and incubated at 37°C for 4 h. After this incubation period, the sensor was washed out before treatment. For IKE treatment, cells were treated with IKE (2 µM), with control wells receiving 0.1% DMSO. Immediately placed on a microscope for imaging as the 0 h time point. For TAM treatment, cells were treated with TAM (2 µM), while control wells were treated with 0.1% DMSO. Samples were imaged at 24 h post-TAM treatment (defined as the 0 h time point). Images were taken using a time-lapse confocal microscope (Zeiss LSM980). Imaging was performed at 8-hour intervals for a duration of 72 hours.

**Immunoblot quantification**

Immunoblot band intensities were quantified using ImageJ. For each sample, the integrated density of the target-protein band was measured after background subtraction and normalized to the corresponding loading control, β-actin or GAPDH, as indicated. Normalized values were then expressed relative to the control group, which was set to 1. Data are presented as mean ± SD from independent biological replicates.

**Immunofluorescence quantification**

Immunofluorescence images were acquired using identical microscope settings across all experimental groups. Fluorescence intensity was quantified using ImageJ. For each group, multiple non-overlapping fields of view were randomly selected from independent samples. Individual cells were manually outlined, and mean fluorescence intensity was measured after background subtraction. The mean intensity of all analyzed cells within each field was calculated, and the average field-level value for each independent sample was used as one biological replicate for statistical analysis. Data are presented as mean ± SD.

**Immunohistochemistry quantification**

Immunohistochemical staining was quantified using ImageJ. For each sample, multiple representative regions were analyzed under identical imaging conditions. Positive staining was identified using a fixed threshold applied consistently across all images. After background correction, either the percentage of positively stained area or the integrated optical density was measured, as appropriate. Measurements from all analyzed regions within each sample were averaged, and the resulting sample-level value was used for statistical analysis. Data are presented as mean ± SD.

**Section 3: Supplementary** **References**

1.	Wang T, Birsoy K, Hughes NW, Krupczak KM, Post Y, Wei JJ*, et al.* Identification and characterization of essential genes in the human genome. Science **2015**;350:1096-101

2.	Ran FA, Hsu PD, Wright J, Agarwala V, Scott DA, Zhang F. Genome engineering using the CRISPR-Cas9 system. Nat Protoc **2013**;8:2281-308

3.	Slyper M, Porter CBM, Ashenberg O, Waldman J, Drokhlyansky E, Wakiro I*, et al.* A single-cell and single-nucleus RNA-Seq toolbox for fresh and frozen human tumors. Nature Medicine **2020**;26:792-802

4.	Hao Y, Hao S, Andersen-Nissen E, Mauck WM, 3rd, Zheng S, Butler A*, et al.* Integrated analysis of multimodal single-cell data. Cell **2021**;184:3573-87 e29

5.	Schupp JC, Adams TS, Cosme C, Raredon MSB, Yuan Y, Omote N*, et al.* Integrated Single-Cell Atlas of Endothelial Cells of the Human Lung. Circulation **2021**;144:286-302

6.	Flodby P, Li C, Liu Y, Wang H, Rieger ME, Minoo P*, et al.* Cell-specific expression of aquaporin-5 (Aqp5) in alveolar epithelium is directed by GATA6/Sp1 via histone acetylation. Scientific Reports **2017**;7:3473

7.	Marconett CN, Zhou B, Sunohara M, Pouldar TM, Wang H, Liu Y*, et al.* Cross-Species Transcriptome Profiling Identifies New Alveolar Epithelial Type I Cell-Specific Genes. Am J Respir Cell Mol Biol **2017**;56:310-21

8.	Katzen J, Beers MF. Contributions of alveolar epithelial cell quality control to pulmonary fibrosis. The Journal of Clinical Investigation **2020**;130:5088-99

9.	Xu Y, Mizuno T, Sridharan A, Du Y, Guo M, Tang J*, et al.* Single-cell RNA sequencing identifies diverse roles of epithelial cells in idiopathic pulmonary fibrosis. JCI Insight **2017**;1

10.	Kimura S, Yokoyama S, Pilon AL, Kurotani R. Emerging role of an immunomodulatory protein secretoglobin 3A2 in human diseases. Pharmacol Ther **2022**;236:108112

11.	He X, Wang L, Riedel H, Wang K, Yang Y, Dinu CZ*, et al.* Mesothelin promotes epithelial-to-mesenchymal transition and tumorigenicity of human lung cancer and mesothelioma cells. Molecular Cancer **2017**;16:63

12.	Liu X, Rowan SC, Liang J, Yao C, Huang G, Deng N*, et al.* Categorization of lung mesenchymal cells in development and fibrosis. iScience **2021**;24:102551

13.	Zhang L, Gao S, White Z, Dai Y, Malik AB, Rehman J. Single-cell transcriptomic profiling of lung endothelial cells identifies dynamic inflammatory and regenerative subpopulations. JCI Insight **2022**;7

14.	Zhang X, Shi X, Xie F, Liu Y, Wei X, Cai Y*, et al.* Dissecting pulmonary fibroblasts heterogeneity in lung development, health and diseases. Heliyon **2023**;9:e19428

15.	Ambhore NS, Kumar A, Sathish V. Molecular determinants of airway smooth muscle cells in health and disease. Ann Transl Med **2022**;10:1084

16.	Huang CH, Schuring J, Skinner JP, Mok L, Chong MMW. MYL9 deficiency is neonatal lethal in mice due to abnormalities in the lung and the muscularis propria of the bladder and intestine. PLoS One **2022**;17:e0270820

17.	Vrhovski B, McKay K, Schevzov G, Gunning PW, Weinberger RP. Smooth Muscle-specific α Tropomyosin Is a Marker of Fully Differentiated Smooth Muscle in Lung. Journal of Histochemistry & Cytochemistry **2005**;53:875-83

18.	Wilting J, Papoutsi M, Christ B, Nicolaides KH, von Kaisenberg CS, Borges J*, et al.* The transcription factor Prox1 is a marker for lymphatic endothelial cells in normal and diseased human tissues. The FASEB Journal **2002**;16:1271-3

19.	Wilson CL, Stephenson SE, Higuero JP, Feghali-Bostwick C, Hung CF, Schnapp LM. Characterization of human PDGFR-β-positive pericytes from IPF and non-IPF lungs. Am J Physiol Lung Cell Mol Physiol **2018**;315:L991-l1002

20.	Pagani F, Tratta E, Dell'Era P, Cominelli M, Poliani PL. EBF1 is expressed in pericytes and contributes to pericyte cell commitment. Histochem Cell Biol **2021**;156:333-47
